# YKL-40 in the brain and cerebrospinal fluid of neurodegenerative dementias

**DOI:** 10.1186/s13024-017-0226-4

**Published:** 2017-11-10

**Authors:** Franc Llorens, Katrin Thüne, Waqas Tahir, Eirini Kanata, Daniela Diaz-Lucena, Konstantinos Xanthopoulos, Eleni Kovatsi, Catharina Pleschka, Paula Garcia-Esparcia, Matthias Schmitz, Duru Ozbay, Susana Correia, Ângela Correia, Ira Milosevic, Olivier Andréoletti, Natalia Fernández-Borges, Ina M. Vorberg, Markus Glatzel, Theodoros Sklaviadis, Juan Maria Torres, Susanne Krasemann, Raquel Sánchez-Valle, Isidro Ferrer, Inga Zerr

**Affiliations:** 1 Network Center for Biomedical Research in Neurodegenerative Diseases (CIBERNED), Institute Carlos III, Ministry of Health, Feixa Llarga s/n, 08907 L’Hospitalet de Llobregat, Barcelona, Spain; 2 Department of Neurology, University Medical School, Göttingen, Germany; 3 German Center for Neurodegenerative Diseases (DZNE), Göttingen, Germany; 4 Laboratory of Pharmacology, School of Health Sciences, Department of Pharmacy, Aristotle University of Thessaloniki, Thessaloniki, Greece; 5 German Center for Neurodegenerative Diseases (DZNE), Bonn, Germany; 6 Bellvitge University Hospital-IDIBELL, Department of Pathology and Experimental Therapeutics, University of Barcelona, Hospitalet de Llobregat, Spain; 7 European Neuroscience Institute, Göttingen, Germany; 8 Institut National de la Recherche Agronomique/Ecole Nationale Vétérinaire, Toulouse, France; 9 Centro de Investigación en Sanidad Animal (CISA-INIA), Madrid, Spain; 10 Institute of Neuropathology, University Medical Center Hamburg-Eppendorf, Hamburg, Germany; 11 Alzheimer’s Disease and Other Cognitive Disorders Unit, Neurology Department, Hospital Clínic, Institut d’Investigacions Biomediques August Pi i Sunyer (IDIBAPS), Barcelona, Spain; 12 Present address: Unit of Lymphoid Malignancies, Division of Experimental Oncology, San Raffaele Scientific Institute, Milan, Italy

**Keywords:** Chitinase 3-like 1, YKL-40, Neuroinflammation, Cerebrospinal fluid, Neurodegenerative dementias, Brain

## Abstract

**Background:**

YKL-40 (also known as Chitinase 3-like 1) is a glycoprotein produced by inflammatory, cancer and stem cells. Its physiological role is not completely understood but YKL-40 is elevated in the brain and cerebrospinal fluid (CSF) in several neurological and neurodegenerative diseases associated with inflammatory processes. Yet the precise characterization of YKL-40 in dementia cases is missing.

**Methods:**

In the present study, we comparatively analysed YKL-40 levels in the brain and CSF samples from neurodegenerative dementias of different aetiologies characterized by the presence of cortical pathology and disease-specific neuroinflammatory signatures.

**Results:**

YKL-40 was normally expressed in fibrillar astrocytes in the white matter. Additionally YKL-40 was highly and widely expressed in reactive protoplasmic cortical and perivascular astrocytes, and fibrillar astrocytes in sporadic Creutzfeldt-Jakob disease (sCJD). Elevated YKL-40 levels were also detected in Alzheimer’s disease (AD) but not in dementia with Lewy bodies (DLB). In AD, YKL-40-positive astrocytes were commonly found in clusters, often around β-amyloid plaques, and surrounding vessels with β-amyloid angiopathy; they were also distributed randomly in the cerebral cortex and white matter. YKL-40 overexpression appeared as a pre-clinical event as demonstrated in experimental models of prion diseases and AD pathology.

CSF YKL-40 levels were measured in a cohort of 288 individuals, including neurological controls (NC) and patients diagnosed with different types of dementia. Compared to NC, increased YKL-40 levels were detected in sCJD (*p* < 0.001, AUC = 0.92) and AD (*p* < 0.001, AUC = 0.77) but not in vascular dementia (VaD) (*p* > 0.05, AUC = 0.71) or in DLB/Parkinson’s disease dementia (PDD) (*p* > 0.05, AUC = 0.70). Further, two independent patient cohorts were used to validate the increased CSF YKL-40 levels in sCJD. Additionally, increased YKL-40 levels were found in genetic prion diseases associated with the *PRNP-*D178N (Fatal Familial Insomnia) and *PRNP-*E200K mutations.

**Conclusions:**

Our results unequivocally demonstrate that in neurodegenerative dementias, YKL-40 is a disease-specific marker of neuroinflammation showing its highest levels in prion diseases. Therefore, YKL-40 quantification might have a potential for application in the evaluation of therapeutic intervention in dementias with a neuroinflammatory component.

**Electronic supplementary material:**

The online version of this article (10.1186/s13024-017-0226-4) contains supplementary material, which is available to authorized users.

## Background

YKL-40 is a glycoprotein secreted by various cell-types including macrophages, chondrocytes, vascular smooth muscle cells and some types of cancer cells [1–4]. In normal adult human tissue higher YKL-40 expression is observed in cells with high cellular activity [5]. Although its precise function is not well understood, it is hypothesized to be involved in tissue remodelling during inflammation and in angiogenic processes mediating infiltration, differentiation and maturation of macrophages, and it is therefore considered a marker of inflammation and endothelial dysfunction [6–9]. Elevated YKL-40 is reported in many pathological conditions such as cancer, diabetes mellitus, cardiovascular disorders and inflammatory diseases of different aetiologies including, among others: bacterial infections, osteoarthritis, hepatic fibrosis, ulcerative colitis, Crohn’s disease, rheumatoid arthritis, asthma, chronic obstructive pulmonary disease and liver cirrhosis [4, 10–14].

In the brain, YKL-40 is upregulated in several neurological disorders such as stroke, lentiviral encephalitis, traumatic brain injury, amyotrophic lateral sclerosis, multiple sclerosis and Alzheimer’s disease (AD) [15–18]. Although YKL-40 was initially associated with the macrophage lineage [19–21], evidence suggests that during neuroinflammatory processes, its expression is abundant in reactive astrocytes and residual in microglial cells [16, 22]. Indeed, astrocytic YKL-40 expression has been reported in both acute and chronic neurological conditions [16] and in close vicinity to amyloid plaques and neurofibrillary tangles in AD [23]. In CSF, YKL-40 is elevated in several acute and chronic neuroinflammatory conditions [16] and in preclinical and prodromal AD/mild cognitive impairment (MCI) [23–26], which is in agreement with the potential role of astrocytosis in early AD pathogenesis [27]. Interestingly, YKL-40 is associated with cerebral morphometric patterns different from those linked to phospho-tau (p-tau)-related neurodegeneration [28], indicating a dissociation between astrocytic activation and p-tau pathology in early AD. Increased CSF YKL-40 levels have been reported in multiple AD cohorts [29–34], and in frontotemporal dementia (FTD) cases [34–36] but not in vascular dementia (VaD) and in DLB/PDD [32, 35], indicating that its deregulation is associated with disease-specific alterations and further suggesting that elevated CSF YKL-40 levels is not a specific signature for AD pathogenesis. However, conflicting data regarding non-AD dementias has been reported [35, 37]. Moreover, absence of studies reporting YKL-40 levels in prion diseases, which present the highest neuroinflammatory component among all dementia conditions [38], impedes establishment of the precise accuracy of YKL-40 quantification in the differential diagnostic context of neurodegenerative dementias. Further, brain YKL-40 expression patterns in different dementia types, and the potential correlation between brain and CSF levels is largely unknown, indicating that more research regarding YKL-40 expression patterns is required. Based on the data presented before, YKL-40 is in the spot light of biomarker research.

In this study, we investigated the sources of YKL-40 in the brain and evaluated its levels in the CSF in different types of neurodegenerative dementias, allowing us to determine the accuracy of YKL-40 quantification in the differential diagnostic context. Our data revealed that YKL-40 expression, while residual in the white matter of control cases, was highly increased in reactive protoplasmic and perivascular astrocytes in sCJD and AD cases. Among the different disease groups studied, highest YKL-40 levels in brain and CSF were observed in sCJD cases, followed by AD and DLB.

## Methods

### Reagents and antibodies

Detection of human YKL-40 by western blot was performed using the Human Chitinase 3-like 1 Antibody AF2599 (R&D Biosystems). Detection of murine YKL-40 by western blot, and human and murine YKL.40 by immunohistochemistry and immunofluorescence was performed using the Chitinase 3-like 1 antibody PA5–43746 (Thermo Fisher).

Anti-GAPDH 9484, anti-β-actin 8226 and anti-vimentin 92547 antibodies were from Abcam. Mouse anti-GFAP MAB360 was from Millipore. Rabbit Anti-GFAP Z0334 and anti-amyloid beta (β-amyloid) M0872 were from Dako. Anti-PrP(12F10) A03221 was from SPI Bio. Thioflavin was from Sigma. Chimeric recombinant prion protein (PrP), composed of the Syrian hamster residues 23–137 followed by sheep residues 141–234 (of the R154 Q171 polymorphism), was from Thermo Fisher.

### Demographics and human cases

Brain tissue was obtained from the Institute of Neuropathology Brain Bank (HUB-ICO-IDIBELL Biobank) and the Biobank of Hospital Clinic-IDIBAPS following relevant guidelines of both Spanish legislation and the local ethics committee. Neuropathological examination and characterization were carried out on paraffin-embedded samples in every case. Detailed information on neuropathology and inflammatory profiling of the sCJD and AD cohorts has been previously reported [39–42]. sCJD MM1 and VV2 cases were selected due to their higher prevalence, but different clinical phenotypes [43]. The presence of infectious, metabolic and neoplastic diseases was ruled out in control samples. No correlation between post-mortem delay or sample storage time and levels of proteins and mRNA analysed was observed. A full summary of the cases is available in Additional file 1.

In order to establish the potential diagnostic utility of CSF YKL-40 measurement in the context of differential diagnosis of neurodegenerative dementias, we utilized samples from three independent cohorts: i) cases recruited at the Clinical Dementia Center at the University Medical Center Göttingen (Germany) (cohort 1), ii) cases recruited at the Hospital Clinic de Barcelona (Spain) (cohort 2) and iii) cases recruited at hospitals of northern Greece that were further processed at the Laboratory of Pharmacology (School of Pharmacy, Aristotle University of Thessaloniki), as part of additional molecular analysis to enhance disease diagnosis (cohort 3). Lumbar puncture was performed for diagnostic purposes followed by analysis of CSF standard parameters. YKL-40, tau, 14–3-3 and Aβ42 were analysed at the point of the first diagnostic work-up. In cohort 1, the neurological control group was composed of patients with either clinically or pathologically defined alternative diagnoses [44, 45]. This group included cases of psychiatric disorders, ischemic stroke, epilepsy, autoimmune diseases, alcohol abuse, headache, vertigo, pain syndromes, acute hypoxia and non-related neurological conditions. Control cases did not present biomarker profiles indicating the presence of a neurodegenerative disease. Diagnosis of AD was based on the International Classification of Diseases, Tenth Revision (ICD-10) definition for AD (F.00-G.30). Rapidly progressive Alzheimer’s disease (rpAD) was defined by a velocity of cognitive decline >6pts/yr. on the Mini Mental Status Examination scale [46]. Velocity of decline was calculated using linear regression (least square method) in accordance with Villemagne et al. [47]. The diagnosis of DLB was based on the criteria of McKeith [17] and PDD as dementia in patients with PD. VaD diagnosis was based on ICD 10 definition (F01) and NINDS-AIREN criteria [48]. In cohort 2, controls were neurologically healthy individuals with no neurological clinical diagnosis and normal neuropsychological assessment. AD patients were diagnosed with probable AD using the National Institute of Neurological and Communicative Disorders and Stroke/Alzheimer’s Disease and Related Disorders Association criteria [49] and displayed the typical CSF biomarker profile, characteristic of AD (low levels of amyloid beta 1–42 (Aβ42) and elevated t-tau and p-tau levels). In cohort 3, controls were composed of subjects with cognitive impairment/dementia (of unknown kind) with excluded prion diagnosis. sCJD cases were classified as definite cases by neuropathological examinations or as probable cases according to diagnostic consensus criteria [50, 51]. Genetic prion cases were confirmed by genetic testing (*PRNP* D178N and E200K mutations). Additional file 2 provides information on demographics and CSF biomarkers results.

### Experimental mouse models

As a sCJD MM1 mouse model, the tg340 mouse line expressing about 4-fold level of human PrP M129 on a mouse PrP null background [52] was used. Control or sCJD MM1 brain tissues were inoculated as 10% (*w*/*v*) homogenates in 6–10 week-old mice in the right parietal lobe using a 25-gauge disposable hypodermic needle. Mice were observed daily and the neurological status was assessed weekly. The animals were sacrificed at pre-symptomatic (pre-clinical: 120 day post inoculation (dpi) and symptomatic (early clinical: 160 dpi and late clinical: 183 dpi) stages. Additionally, sCJD MM1 inoculum dilutions were performed to study prolonged disease times; animals were sacrificed at 210 dpi (10–1 dilution). Part of the brain was fixed by immersion in 10% buffered formalin to quantify spongiform degeneration and perform immunohistological analyses. The remaining brain was snap frozen and stored at −80 °C for protein and RNA studies. The 5xFAD mouse line harbouring the APP KM670/671NL (Swedish), APP I716V (Florida), APP V717I (London), PSEN1 M146 L (A&gt;C), PSEN1 L286 V mutations was used as a model of AD pathogenesis. This widely-used model recapitulates several AD-related phenotypes displaying severe amyloid pathology and gliosis at about 2–3 months, cognitive alterations at 4–5 months and synaptic and neuronal loss at 9 of age [53, 54]. For scrapie infections with prion strains RML (immunohistochemical data) and 22 L (qPCR data), all animal experiments were in strict accordance with the principles of laboratory animal care (NIH publication No. 86–23, revised 1985) and the recommendations in the Guide for the Care and Use of Laboratory Animals of the German Animal Welfare Act on protection of animals. Animals were maintained under specific pathogen-free conditions. Mice were anesthetized with Ketamine/Xylazin hydrochloride prior to inoculation. Eight weeks old mice were inoculated intracerebrally (i.c.) with RML 5.0-prions (immunohistochemical data) and 22 L prions (qPCR data). Animals were checked daily and were taken at indicated time points or were allowed to progress to clinical prion disease (presenting with slowness, ataxia, weight loss, trembling and ungroomed fur). For scrapie infections with RML strain (qPCR data), all experiments were performed in the animal facility of the Neurological Department, AHEPA University Hospital. Approximately 8–12 weeks old mice were inoculated. Infectious material corresponding to 100 μl of 1% brain homogenate from terminally ill RML-infected C57Bl/6 mice (104.5 × LD50) was intraperitoneally administered in fourteen animals; ten age-matched unchallenged mice were used as controls. RML-infected C57Bl/6 mice developed the first clinical symptoms, corresponding to abnormal gait, at approximately 130 days post inoculation. More severe symptoms, including loss of muscle tone, hind limb weakness, ataxia, weight loss and ruffled coat, appeared at approximately 160 days post inoculation. Whole brain was harvested from three animals per group at pre-clinical (120 dpi) and end-point clinical (180 dpi) disease stages.

### Organotypic slice cultures

Organotypic cerebellar slice preparations from postnatal day 9–13 (P9–13) pups of C57BL/6JRj mice, further infection with 22 L scrapie strain, sample collection and detection of PK-resistant Prion Protein (PrPres) were performed as described previously [55].

### CSF analysis

Total tau was measured using INNOTEST™ hTAU Ag; (Fujirebio). Levels of amyloid β1–42 (Aβ42) were measured with an ELISA kit (INNOTEST™ AMYLOID (1–42); Fujirebio). YKL-40 was measured using the MicroVue YKL-40 EIA ELISA kit from Quidel following the manufacturer’s instructions. Protein 14–3-3 was detected with western blot as previously described [56].

### RNA purification and RT-qPCR

RNA from different human and mouse brain regions was purified using the miRVANA RNA isolation kit following the manufacturer’s protocol. For human samples, regions utilized for RNA extraction and corresponding age and gender information is summarized in Additional file 1. RNA was extracted from the following murine brain regions: tg340MM1-CJDMM1: cerebral cortex, RML scrapie: whole brain, 22 L scrapie: cerebral cortex and cerebellum. RNAs were treated with DNase Set (Qiagen) to eliminate genomic DNA contamination. Sample concentration was measured using a NanoDrop 2000 spectrophotometer (Thermo Scientific) and RNA integrity was assessed based on the RNA Integrity Number (RIN value) measured by an Agilent 2100 Bioanalyzer (Agilent). RNA samples were retrotranscribed using the High Capacity cDNA Archive kit (Applied Biosystems). Quantitative PCR assays were performed in duplicates on cDNA samples, in a LightCycler® 480 System (Roche). The reactions were set using 20xTaqMan Gene Expression Assays and 2xTaqMan Universal PCR Master Mix (Applied Biosystems) and conducted under the following conditions: 50 °C for 2 min, 95 °C for 10 min, 40 cycles at 95 °C for 15 s and 60 °C for 1 min. Fold changes were estimated using the 2-ΔΔCT equation. Fold changes in mRNA expression were determined relative to the control cases after normalization with housekeeping genes; statistical significances were calculated using GraphPad Prism 6.01. As housekeeping genes, *GAPDH* and *GUSB* as well as G*usb* and *Hprt* were used as in human and mouse samples respectively. The Taqman probes used were: CHI3L1 (YKL-40) human: Hs01072228_m1, GFAP human: Hs00909240_m1, AIF1 (IBA-1) human: Hs00741549_g1, GAPDH human: Hs03929097_g1, GUSB human: Hs00939627_m1, Chi3l1 (YKL-40) mouse: Mm00801477_m1, Hprt mouse: Mm03024075_m1, GusB mouse: Mm00446958_g1.

### Western blotting

Human and mouse tissues were lysed in lysis buffer containing: 100 mM Tris pH 7, 100 mM NaCl, 10 mM EDTA, 0.5% NP-40 and 0.5% sodium deoxycolate plus protease and phosphatase inhibitors (Sigma). Samples were centrifuged (14,000×g for 20 min at 4 °C) and supernatants were quantified for protein concentration using the Bradford method (Biorad). Samples were mixed with SDS-PAGE sample buffer, heated to 95 °C for 10 min, and resolved by SDS-PAGE analysis. Gels were transferred onto PVDF membranes, blocked and incubated with anti-YKL-40 antibodies (1:1000 dilution) as indicated above. Following incubation with HRP-conjugates, species-specific secondary antibody, the membranes were processed for specific immunodetection using the ECL reagent. GAPDH and β-actin antibodies at 1:5000 dilutions were used as loading controls. Fold changes (in arbitrary units) were determined from densitometric analysis relative to the control cases.

### Immunohistochemistry and immunofluorescence

The immunohistochemical study was performed on 4-μm thick dewaxed brain sections. Endogenous peroxidases were blocked with peroxidase (Dako, Glostrup) followed by 10% normal goat serum. Following incubation with the anti-YKL-40 antibody (diluted 1:200) at room temperature overnight, the sections were incubated with EnVision1 system peroxidase (Dako) for 15 min at room temperature. The peroxidase reaction was visualized with diaminobenzidine (DAB) and H_2_O_2_. Primary antibody was omitted in some sections as immunostaining control and no signal was detected after incubation with the secondary antibody. Sections were counterstained with haematoxylin. For immunofluorescence, 4 μm thick dewaxed sections were stained with a saturated solution of Sudan black B (Merck, Germany) for 15 min to block the autofluorescence of lipofuscin granules present in cell bodies, and then rinsed in 70% ethanol and washed in distilled water. The sections were boiled in citrate buffer to enhance antigenicity, blocked for 30 min at room temperature with 10% fetal bovine serum diluted in phosphate buffered saline, and incubated at 4 °C overnight with primary antibodies. Double-labelling immunofluorescence was performed using a combination of YKL-40 (1:100) and GFAP (1:200 or 1:400) antibodies or PrP (12F10) (1:200) and either Aβ (1:50), YKL40 (1:100) or GFAP (1:400) antibodies. After washing, the sections were incubated with Alexa (Molecular Probes) fluorescence secondary antibodies (Alexa-Fluor 488 and 555 conjugated secondary antibodies). Nuclei were stained with DRAQ5™ (Biostatus Ltd., Shepshed, UK). After washing, the sections were mounted in Immuno-Fluore mounting medium (ICN Biomedicals, Irvine, CA), sealed and dried overnight. Confocal images were acquired using a microscope Leica DMIRE2 and Leica confocal software. Z-Stack image interval was 0.5 μm. Single plane images for quantification were obtained by Nikon Eclipse E800 microscope with ProgRes® CapturePro 2.7.7 software (JENOPTIK). Quantification of YKL-40 and/or GFAP positive cells was performed manually using Fiji ImageJ software in 13–15 areas per section in 3 different patients.

Immunofluorescence of frozen brain sections in 5xFAD mice was carried out as described [57]. For the analysis of human samples, two to four cases per diagnostic group were used and three to four sections from every case were analysed. For immunohistochemical analysis upon prion infection, tissues were fixed with 4% buffered formalin, prion infected samples were inactivated by immersion in 98% formic acid for one hour, post fixed in 4% buffered formalin overnight and processed for paraffin embedding. Sections (2 μm) were subjected to staining with the CHI3L1 antibody (YKL-40, 1:100) using a Ventana Benchmark XT (Ventana, Tuscon, Arizona, USA). Sections were incubated with primary antibody for 1 h, anti-rabbit Histofine Simple Stain MAX PO Universal immunoperoxidase polymer (Nichirei Biosciences, Wedel, Germany) was used as secondary antibody. Detection of secondary antibodies and counter staining was performed with an ultraview universal DAB detection kit from Ventana (Ventana, Tuscon, Arizona, USA). Representative images were taken with a Leica DMD108 digital microscope.

### Elisa

Detection of murine YKL-40 in tissue homogenates was performed using the Mouse Chitinase 3-like 1/YKL-40 PicoKine™ ELISA Kit (Bosterbio) following the manufacturer’s instructions. Inter- and intra assay coefficients of variation were below 15%. Dilution linearity tested from neat CSF to 1:8 dilution factor. Percent linearity was 85–112%. For the assays, CSF was diluted 1:3.

### Real-time quaking induced conversion (RT-QuIC)

RT-QuIC was performed as described previously [58, 59] with minor modifications. Briefly, 85 μl of reaction buffer containing recombinant PrP (10 μg) was mixed with 15 μl of a 1:100 dilution of 10%*w*/*v* brain homogenates. Tissues for RT-QuIC analysis were lysed in PBS 0.1% SDS and clarified by centrifugation for 10 min at 10000×g. Reactions were set at a final volume of 100 μl and placed in 96-well black optical bottom plates (Fisher Scientific). Each sample was run in triplicate. Plates were sealed and incubated in a FLUO Star OPTIMA plate reader (BMG Labtech Ortenberg, GE) at 42 °C for 80 h with intermittent shaking cycles, consisting of 1 min double orbital shaking at the highest speed (600 rpm) followed by a 1 min break.

### Statistical analysis

For two-group comparisons the *Mann–Whitney* test (non-parametric distribution) and unpaired t-test (parametric distribution) were used. In multiple comparisons, the *Kruskal–Wallis* test was used. Dunn’s multiple comparison test was used for post-hoc analysis. Values of *p* < 0.05 were considered significant. *Pearson* (parametric distribution) and *Spearman* (non-parametric distribution) tests were used to detect correlations between biomarkers, demographic data and mRNA expression levels. Two-way ANOVA followed by Bonferroni’s post-hoc was used in the quantification YKL-40 and GFAP positive cells. Receiver operating characteristic (ROC) curves and derived area under the curve (AUC; AUC = 0.5 no discrimination and AUC = 1 perfect discrimination) were calculated. The best cut-off value, sensitivity and specificity were estimated based on the *Youden* index [60] (point on an ROC curve providing the best balance of both sensitivity and specificity). ROC curves and statistical analyses were performed using the Graph Pad Prism 6.01 software.

### Genetic testing

For detection of a prion disease-associated mutation, genetic testing was performed on genomic DNA isolated from blood or brain tissue [61]. Informed consent for the prion protein gene (*PRNP*) analysis was obtained from each patient or legal guardian.

## Results

### YKL-40 expression in sCJD patients

**Fig. 1:**
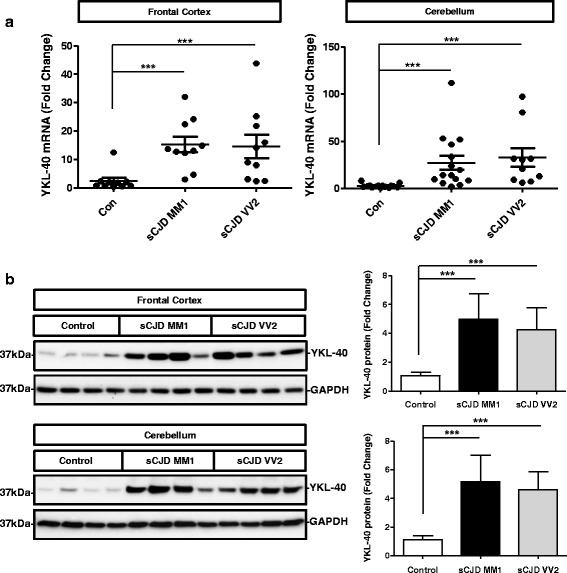
YKL-40 expression in the brain tissue of sCJD and related mouse model. **a** RT-qPCR analysis of YKL-40 in the frontal cortex (left panel) and cerebellum (right panel) of control, sCJD MM1 and sCJD VV2 samples. GAPDH was used for normalization. **b** Western blot analysis of YKL-40 in the frontal cortex (upper panel) and cerebellum (bottom panel) of control, sCJD MM1 and sCJD VV2 samples. For normalization GAPDH was used. Graphical representation of Western Blot data acquired from the analysis of eight samples per group. Fold changes in the expression of mRNA and protein were determined relative to the control cases. Kruskal-Wallis and Dunn’s post-hoc tests were used to determine statistical differences. ***p < 0.001

Expression of YKL-40 was initially analysed in sCJD as this neurodegenerative dementia presents the highest neuroinflammatory profile, including massive gliosis [39] and regulation of inflammatory mediators [38] compared to other neurodegenerative dementias [38, 40]. mRNA analysis showed that YKL-40 was highly increased in the frontal cortex (FC) and cerebellum (CB) of sCJD subtypes MM1 and VV2 (*p* < 0.001). No statistically significant differences were observed between sCJD subtypes in the two regions studied. Mean fold change values for controls versus sCJD comparisons were 6.1 in the FC and 11.5 in the CB (Fig. 1a). YKL-40 levels were normalized with GAPDH, and similar results were obtained for GUSB normalization (Additional file 3A). In agreement with mRNA data, YKL-40 protein levels were increased in the FC and CB of sCJD subtypes MM1 and VV2 (*p* < 0.001). Mean fold change values for controls versus sCJD comparisons were 4.5 in the FC and 4.6 in the CB (Fig. 1b).

**Fig. 2:**
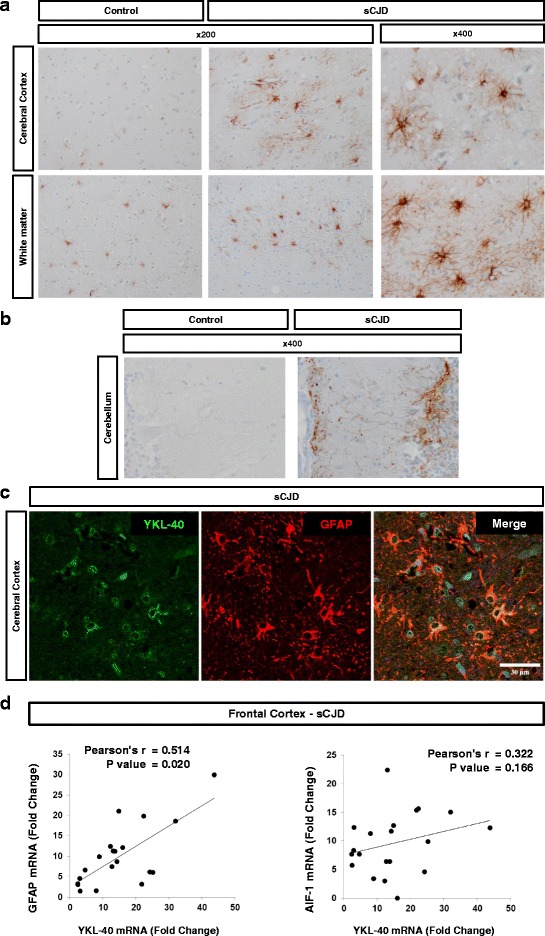
YKL-40 expression in sCJD brain tissue. **a** Immunohistochemical analysis for detection of YKL-40 in the cerebral cortex and white matter of control and sCJD cases. **b** Immunohistochemical analysis of YKL-40 in the cerebellum of control and sCJD cases. Brown staining corresponds to YKL-40 staining and light blue to haematoxylin counterstaining. **c** Immunofluorescence analysis of YKL-40 (green) and GFAP (red) in cerebral cortex region of sCJD. Nuclei were stained with DAPI (blue). **d** Correlation between the expression levels of YKL-40 and GFAP mRNA (left panel) or AIF-1 mRNA (right panel) in the frontal cortex of sCJD cases. Normalization was performed using GAPDH. Pearson test was used to determine the correlations between mRNA expression levels

Further, immunohistochemistry was used for the study of YKL-40 expression and distribution in control and sCJD brains. YKL-40 immunoreactivity in normal brain was restricted to fibrillar astrocytes (stellate astrocytes with long processes) in the white matter and was increased in both the cerebral cortex and white matter of sCJD brain tissue sections compared to controls (Fig. 2a). YKL-40 was localized predominately in reactive protoplasmic and perivascular astrocytes, in addition to increased signal in fibrillar astrocytes of the white matter. Increased YKL-40 immunoreactivity was also detected in the Bergmann glia in the CB of sCJD cases (Fig. 2b). No apparent correlation was observed between PrPSc amorphous deposits in the CB in VV2 and YKL-40-positive astrocytes (data not shown). Specific expression of YKL-40 in astrocytes was confirmed by a positive co-localization between YKL-40 and GFAP in FC (Fig. 2c). Interestingly, double PrP-GFAP and PrP-YKL-40 immunostaining in the FC of sCJD revealed the presence of astrocytic processes within the PrP plaque-like deposits (Additional file 4A and 4B).

Additionally, we found a positive correlation between mRNA levels of YKL-40 and the astrocytic marker GFAP (*p* < 0.01) in the FC of sCJD cases, while no correlation was observed with the microglial marker AIF1 (*p* > 0.05) (Fig. 2d). As positive but moderate correlation between GFAP and YKL-40 expression levels were observed, the association between YKL-40+ and GFAP+ astrocytes was further explored. Quantification of double-labelled GFAP and YKL-40 immunoreactive cells showed co-localization in a majority of cells (GFAP+/YKL-40+ = 251 ± 8 cells/mm2) whereas the number of cells showing only GFAP staining or only YKL-40 staining was significantly smaller (44 ± 15 cells/mm2 and 3 ± 2 cells/mm2, respectively (*p* < 0.001) (Additional file 5A and B).

### YKL-40 expression in in vivo experimental models of prion disease

**Fig. 3:**
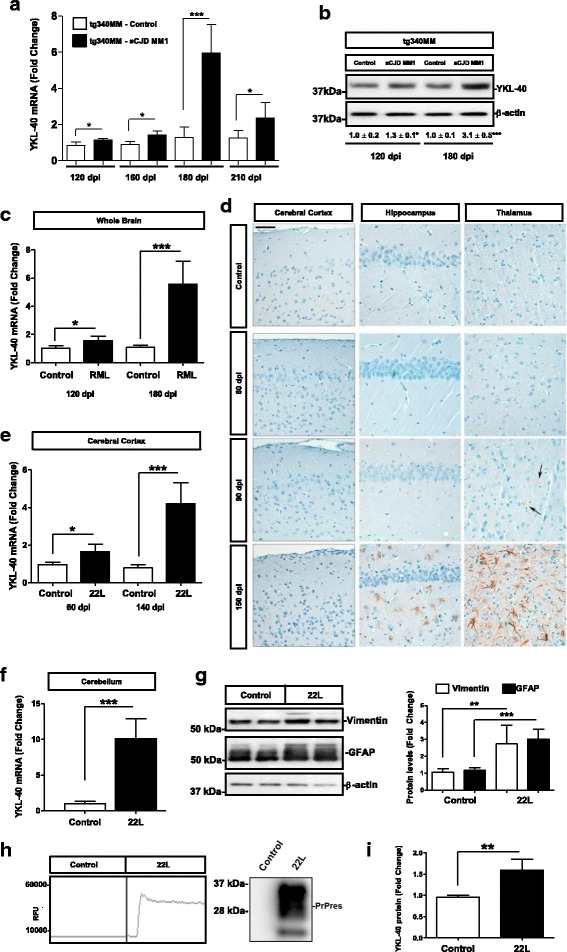
YKL-40 expression in experimental models of prion diseases. **a** RT-qPCR analysis of YKL-40 in the cortex of control and sCJD MM1 inoculated tg340*PRNP*129MM mice at 120 dpi (pre-clinical), 160 dpi (early clinical), 180 dpi (clinical) and 210 dpi (clinical with 10–1 diluted inoculum). Four animals per group were analyzed. Normalization was performed using Hprt. **b** Representative Western-blot analyses for YKL-40 immunodetection in the cortex of control and sCJD MM1-inoculated tg340*PRNP*129MM mice at 120 dpi (pre-clinical) and 180 dpi (clinical). Three animals per group were analyzed. Normalization was based on β-actin levels. Numbers indicate densitometry results from three animals per group. Unpaired t-tests were performed to determine statistical differences. **c** RT-qPCR analysis of YKL-40 in the whole brain of control and RML scrapie-infected mice at pre-clinical (120 dpi) and clinical disease (180 dpi) stages. GAPDH was used for normalization. Similar results were acquired when normalization was based on Hprt expression levels (not shown). Unpaired t-tests were used for estimation of statistical differences. **d** Immunohistochemical analysis of YKL-40 expression in the cerebral cortex, hippocampus and thalamus of control and RML scrapie-infected mice at pre-clinical (60 and 90 dpi) and clinical (150 dpi) disease stages. Scale bar = 50 μm. Arrows indicate YKL-40 positive reactive astrocytes. Three animals per time point were used. Two sections were stained per animal (sagittal and coronal sections were used). Brown staining corresponds to YKL-40 staining and light blue to haematoxylin counterstaining. **e** RT-qPCR analysis of YKL-40 in the cerebral cortex of control and 22 L scrapie-infected mice at pre-clinical (60 dpi) and clinical (140 dpi) disease stages. **f** RT-qPCR analysis of YKL-40 in the cerebellum of control and 22 L scrapie-infected mice at clinical (140 dpi) disease stages. In all cases GAPDH was used for normalization. Similar results were obtained when Hprt was used for normalization (not shown). Unpaired t-tests were used to determine statistical differences. **g** Western blot analysis of Vimentin and GFAP in control and 22 L–scrapie infected COCS. β-actin was used for normalization. Graphic representation of densitometry analysis of five cerebellar tissues per condition is shown. Statistical differences were determined with unpaired t-tests. **h** RT-QuIC analysis of control and 22 L–scrapie infected COCS. A representative graph of three cerebellar tissues per condition is shown. All control tissues were tested negative, while all 22 L–infected tissues were tested positive (left panel); western-blot showing the presence of PrPres in 22 L–infected cells (right panel). **i** ELISA analysis of YKL-40 levels in control and 22 L–scrapie infected COCS. Statistical differences were determined with unpaired t-tests. Fold changes in the expression of mRNA and protein were determined relative to the control cases. *p < 0.05, **p < 0.01, ***p < 0.001

To gain insight into the expression levels of YKL-40 in pre-clinical and early clinical sCJD stages, we took advantage of the tg340-*PRNP*-129MM mouse line inoculated with sCJD MM1 brain homogenate, which fully recapitulates the neuroinflammatory profile of human patients [40]. Analysis of the mRNA levels indicated that YKL-40 was increased at pre-clinical (120 dpi) and early-clinical (160 dpi) disease stages (*p* < 0.05). Differences between control and sCJD MM1-inoculated animals were more significant at clinical stages (180 dpi, *p* < 0.001 and 210 dpi, *p* < 0.01), resembling observations in sCJD post-mortem brains (Fig. 3a). Western blots from the same set of inoculated animals validated the presence of elevated YKL-40 at pre-clinical (*p* < 0.05) and clinical (*p* < 0.001) disease stages at the protein level as well (Fig. 3b).

To ascertain whether increased YKL-40 expression was also present in non-human prion diseases, wild-type mice inoculated with 22 L and RML scrapie strains were utilized. Increased YKL-40 mRNA levels were detected in the whole brain of RML-infected animals at late pre-clinical (120 dpi, *p* < 0.05) and clinical (180 dpi, *p* < 0.001) stages of the disease (Fig. 3c). Immunohistochemical analysis of RML-infected mice revealed the presence of YKL-40 positive astrocytes in the hippocampus and thalamus at clinical stages. In contrast to sCJD infected mice no staining was detected in cortical regions (Fig. 3d). At pre-clinical stages sparse positive YKL-40 positive cells were detected in the thalamic nuclei (Fig. 3d). In contrast, mice inoculated with the 22 L strain, which led to a faster clinical onset and a distinct neuropathology including more cortical PrPSc deposition pattern, presented increased YKL-40 mRNA levels in the cerebral cortex at pre-clinical (*p* < 0.05) and clinical (*p* < 0.001) disease stages (Fig. 3e). Additionally, YKL-40 mRNA levels were highly increased in the cerebellum of 22 L infected mice (Fig. 3f) (*p* < 0.001).

### YKL-40 expression in ex vivo experimental models of prion disease

The observation of increased YKL-40 in the cerebellum of 22 L infected mice allowed to speculate that (22 L)-infected cultured organotypic cerebellar slices (COCS), which recapitulate most of the morphological and neuropathological hallmarks associated with prion infection and serve as an excellent ex vivo tool for the study of prion pathogenesis [55, 62], could recapitulate the alterations in YKL-40 expression observed in in vivo scrapie models. Ten weeks post-22 L infection, we detected astrogliosis by means of vimentin and GFAP expression (Fig. 3g). This was accompanied by positive seeding ability of pathogenic PrP by means of RT-QuIC analysis and presence of PrPres demonstrating the presence of prion infection on the COCS (Fig. 3h). At this time point, increased YKL-40 protein was detected in scrapie-infected COCS compared to mock treated cells (*p* < 0.01) (Fig. 3i).

### YKL-40 expression in AD patients and in AD experimental model

**Fig. 4:**
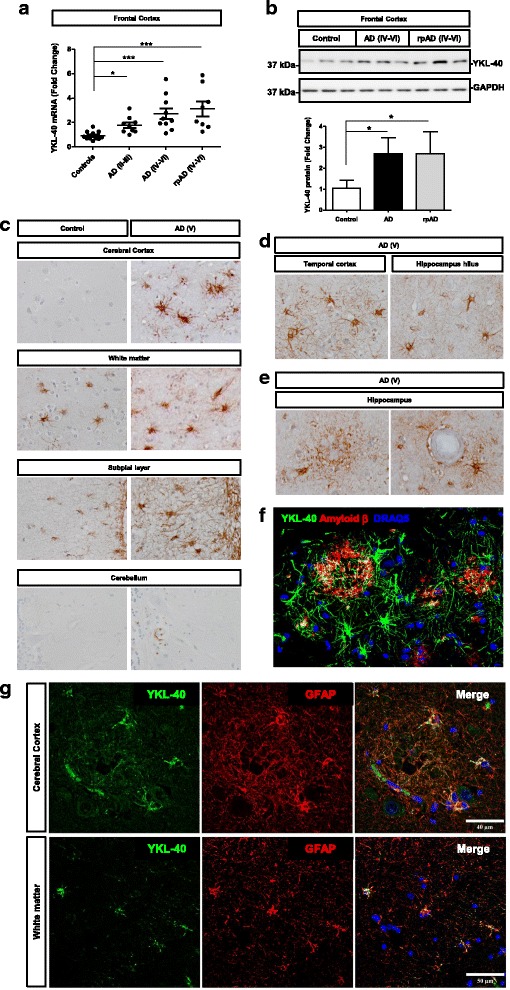
YKL-40 expression in AD brain tissue (**a**) RT-qPCR analysis of YKL-40 in the frontal cortex of control, AD (I-III), AD (IV-VI) and rpAD (IV-VI) samples. GAPDH was used for normalization. Kruskal-Wallis and Dunn’s post-hoc tests were used to estimate statistical differences. **b** Western blot analysis of YKL-40 in the frontal cortex of control, AD (IV-VI) and rpAD (IV-VI) samples. Normalization was based on GAPDH levels. Graphic summary of densitometry analyses performed on western blot results acquired from 8 control, 8 AD and 6 rpAD samples. **c** Immunohistochemical analysis of YKL-40 in the cerebral cortex, white matter, subpial layer and cerebellum in control and AD cases. **d** Immunohistochemical analysis of YKL-40 in the temporal cortex and hippocampus in AD cases. **e** Immunohistochemical analysis of YKL-40+ astrocytes surrounding β-amyloid plaques (left) and in blood vessels with amyloid angiopathy (right) in the hippocampal region of AD cases Brown staining corresponds to YKL-40 staining and light blue to haematoxylin counterstaining. **f** Double-labeling immunofluorescence of YKL-40 (green) and amyloid β (red) in the hippocampus of AD. **g** Double-labeling immunofluorescence of YKL-40 (green) and GFAP (red) in cerebral cortex and white matter in AD tissues. Fold changes in expression of mRNA and protein were determined relative to the control cases. *p < 0.05, ***p < 0.001

Compared to controls, YKL-40 mRNA levels were increased in the FC of AD cases at early (AD I-III, *p* < 0.05) and late (AD IV-VI and rpAD IV-VI, *p* < 0.001) neurofibrillary tangle Braak stages (Fig. 4a). Mean fold change values for controls versus AD comparisons were 1.8 for AD II-III and, 2.9 for AD IV-VI and 3.2 for rpAD IV-VI. No differences were detected between AD and rpAD cases (*p* < 0.05). YKL-40 mRNA levels were normalized with GAPDH, and similar results were obtained for GUSB normalization (Additional file 3B). In agreement with mRNA data, YKL-40 expression at protein level was increased in the FC of AD IV-VI (fold change 2.58) and rpAD IV-VI (fold change 2.59) cases (*p* < 0.05) (Fig. 4b).

Moreover, YKL-40 expression was analysed by immunohistochemical staining in several AD brain regions. Increased YKL-40 expression was detected in the cerebral cortex, white matter and subpial layer of AD sections compared to controls, while it remained unchanged in the cerebellum of AD cases (Fig. 4c). Increased expression was also detected in the temporal cortex and throughout the hippocampus region of AD brain sections (Fig. 4d, and Additional file 6A). YKL-40 staining was mainly detected in astrocytes, similarly to the patterns observed in sCJD cases. In contrast with the diffuse distribution of YKL-40-immunoreactive astrocytes in sCJD, YKL-40-positive astrocytes in the cerebral cortex in AD predominated in clusters often surrounding a core of fibrillar β-amyloid (Fig. 4e). YKL-40-positive astrocytes were also present around blood vessels with β-amyloid (Fig. 4e and Additional file 6B). Double-labelling with YKL-40 and β-amyloid antibodies was used to demonstrate the presence of YKL-40 positive astrocytes surrounding senile and diffuse β-amyloid plaques in the hippocampus of AD cases (Fig. 4f and Additional file 7).

Fibrillar astrocytes in the subcortical white matter and deep white matter were also strongly YKL-40 immunoreactive. Specific expression of YKL-40 in GFAP-immunoreactive astrocytes in the cerebral cortex and white matter regions (Fig. 4g) in AD brains was confirmed by double-labelling immunofluorescence and confocal microscopy. Quantitative assessment of double-labelled GFAP and YKL-40 cells indicated a significant superior number of astrocytes showing co-localization of GFAP and YKL-40 (238 ± 23 cells/mm2), compared to those showing only GFAP or only YKL-40 staining (28 ± 6 cells/mm2 and 17 ± 3 cells/mm2, respectively) (*p* < 0.001) (Additional file 5C and D).

As an in vivo model of AD pathogenesis we used the 5xFAD mouse model. At 3 months of age, 5xFAD animals presented glial changes and amyloid plaques but do not show clinical signs or behavioural alterations [53, 54]. At this time point, we found increased YKL-40 and GFAP protein levels (*p* < 0.05) (Additional file 8A). At immunohistochemical level, widespread astrocytic YKL-40 expression was detected in 10-month-old 5xFAD animals, especially in the cerebral cortex and hippocampus (Additional file 8B) as well as in the thalamus, caudate putamen and midbrain, while sparse YKL-40 positive astrocytes could be detected in the molecular layer of cerebellum. At this stage, animals showed clear clinical and behavioural signs.

### YKL-40 in DLB

**Fig. 5:**
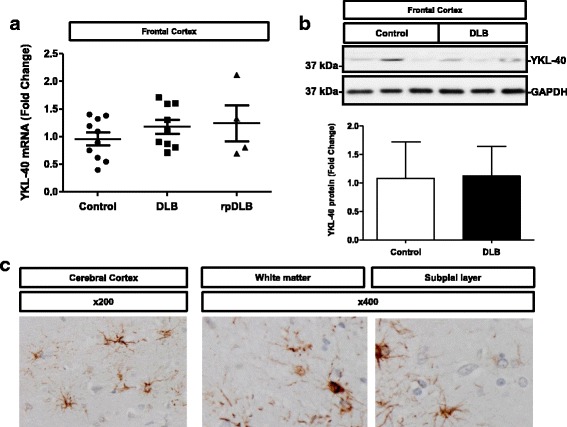
YKL-40 expression in DLB brain tissue. **a** RT-qPCR analysis of YKL-40 in the frontal cortex of control, DLB and rpDLB samples. Normalization was based on GAPDH levels. **b** Western blot analysis of YKL-40 in the frontal cortex of control, DLB and rpDLB samples. Normalization was carried out with GAPDH. Kruskal-Wallis and Dunn’s post-hoc tests were used for determination of statistical differences. **c** Immunohistochemical analysis of YKL-40 in the cerebral cortex, white matter and subpial layer in DLB cases. Brown staining corresponds to YKL-40 staining and light blue to haematoxylin counterstaining. Fold changes in expression (mRNA and protein) were determined relative to the control cases

YKL-40 mRNA expression was further analysed in the FC of DLB cases. Despite increased mean values of YKL.40 detected in DLB and rapid forms of DLB (rpDLB) compared to controls, no statistically significant alterations were found between these groups (Fig. 5a). Similarly, no differences at the protein level were detected between control and DLB cases (Fig. 5b). Sparse YKL-40-positive astrocytes were observed in the cortical regions, white matter and subpial layer in DLB cases; sparse clusters of YKL-40-positive astrocytes were not uncommon due to the fact of the associated low burden of AD-related pathology in DLB (Fig. 5c).

### CSF YKL-40 in the differential diagnosis of neurodegenerative dementias

**Fig. 6:**
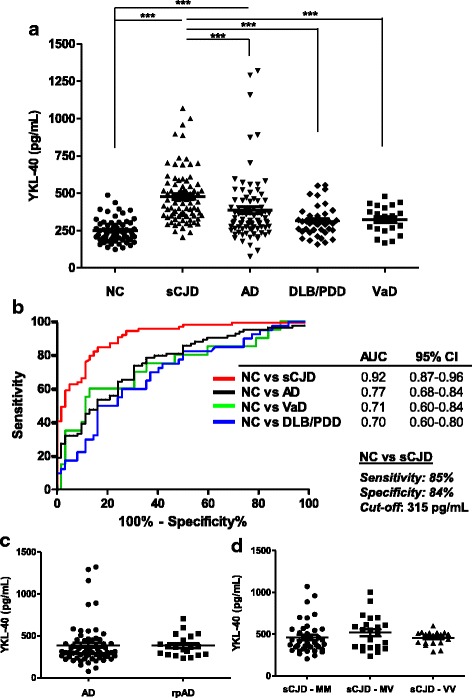
CSF YKL-40 in the differential diagnosis of neurodegenerative dementia. **a** CSF YKL-40 in NC (*n* = 62), sCJD (*n* = 84), AD (*n* = 84), DLB/PDD (*n* = 40) and VaD (*n* = 20) cases (cohort 1). Number of cases analyzed and statistical significance of differences between groups is indicated. Kruskal-Wallis and Dunn’s post-hoc tests were used for estimation of statistical differences. **b** ROC curves for YKL-40 quantification in the differential diagnosis of dementia groups compared to NC. In the legend, AUC values, corresponding to the area under ROC curves, and 95% confidence intervals are reported. Diagnostic parameters (cut-off, sensitivity and specificity) are indicated for NC versus sCJD comparison. **c** Stratification of AD cases in slow progressive (AD) (*n* = 64) and rapid progressive (rpAD) (n = 20) cases. Statistical differences were tested with Mann–Whitney test. **d** Stratification of sCJD cases according to *PRNP* codon 129 polymorphism (MM, *n* = 40, MV, *n* = 22 and VV, *n* = 20). Kruskal-Wallis and Dunn’s post-hoc tests were used for statistical differences estimation. ***p < 0.001

When CSF YKL-40 was analysed in the differential diagnostic context of neurodegenerative dementias (cohort 1, Additional file 2), significantly higher YKL-40 levels were detected in sCJD cases (480 ± 178 pg/mL) compared to neurological controls (NC) (250 ± 77 pg/mL) and to the other dementia groups (*p* < 0.001) (Fig. 6a, Additional file 2). Compared to NC, YKL-40 was also increased in AD patients (386 ± 221 pg/mL, *p* < 0.001) but not in VaD (323 ± 96 pg/mL, *p* > 0.05) and DLB/PDD cases (314 ± 103 pg/mL, *p* > 0.05). To calculate the clinical accuracy of YKL-40 in discriminating between dementia and NC groups, we estimated the AUC values. As expected, the highest values were detected when comparing NC and sCJD cases (AUC: 0.92, 95% CI: 0.87–0.96) (Fig. 6b). Using an optimal cut-off at 315 pg/mL defined by the *Youden* index, an overall sensitivity of 85% and specificity of 84% could be achieved. For the rest of the dementias AUC values were far below those detected for sCJD cases: NC vs AD; AUC: 0.77, 95% CI: 0.68–0.84, NC vs VaD; AUC: 0.71, 95% CI: 0.60–0.84 and NC vs DLB: AUC: 0.70, 95% CI: 0.60–0.80 (Fig. 6b). Next we explored the ability of CSF YKL-40 to discriminate sCJD from the rest of dementia groups. While YKL-40 could not discriminate sCJD from AD with high accuracy (AUC: 0.70, 95% CI: 0.62–0.78), values were higher for sCJD vs DLB/PDD (AUC: 0.81, 95% CI: 0.72–0.89) and sCJD vs VaD (AUC: 0.77, 95% CI: 0.67–0.88) comparisons.

Further we analysed whether YKL-40 levels could be influenced by the rate of cognitive decline and/or disease duration. For this reason, we stratified AD cases into slow and fast progressive AD (AD versus rpAD) and sCJD samples according to codon 129*PRNP* polymorphism, which plays a role in the clinico-pathological phenotype of the disease and, as a consequence, in disease duration [43, 63, 64]. No differences in CSF YKL-40 levels were observed between AD (387 ± 244 pg/mL) and rpAD (384 ± 128 pg/mL) cases or between sCJD cases according to their codon 129 polymorphism: sCJD MM (462 ± 195 pg/mL), MV (519 ± 203 pg/mL) and VV (456 ± 79 pg/mL) (Fig. 6c, d).

**Fig. 7:**
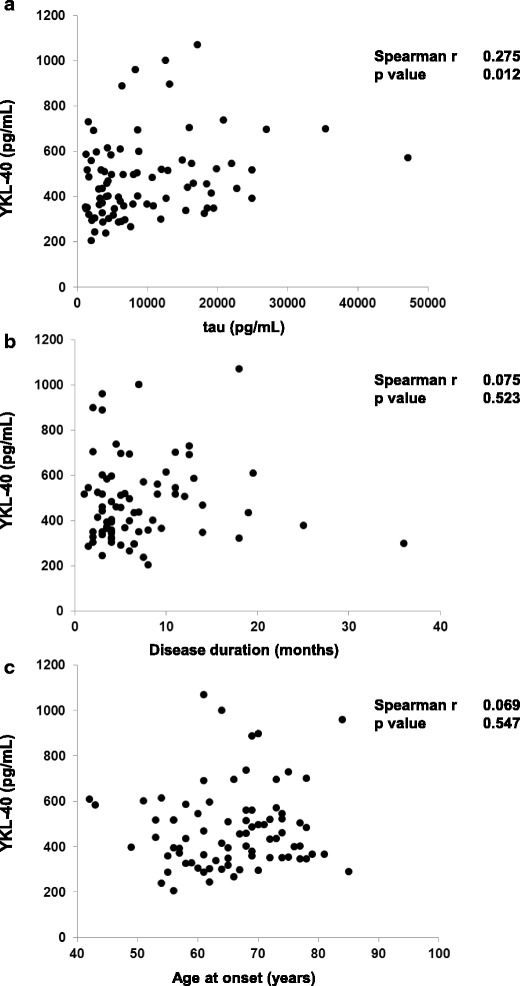
Correlation of CSF YKL-40 levels with CSF tau levels, disease duration and age at onset. Correlation analysis between CSF YKL-40 and (**a**) CSF tau levels, (**b**) disease duration (as time in months from disease onset to death) and (**c**) age at onset. The association between CSF YKL-40 and either biomarkers or demographics parameters was investigated with the Spearman correlation. Positive correlation was detected only for CSF YKL-40 versus CSF tau comparison

Next we sought to investigate the potential correlation between YKL-40 and tau, disease duration and age in sCJD cases. A significant positive correlation of YKL-40 with CSF tau was found (*p* < 0.05) (Fig. 7a), but no correlation of YKL-40 levels with CSF Aβ42, disease duration and age at disease onset was detected (*p* > 0.05) (Fig. 7b and c).

### Validation of increased YKL-40 levels in the CSF of prion diseases

**Fig. 8:**
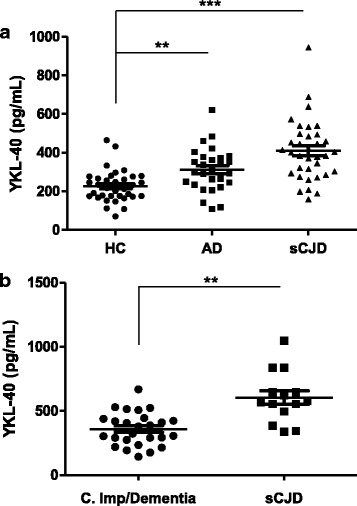
Independent validation of increased CSF YKL-40 level in sCJD patients. **a** CSF YKL-40 levels in HC (*n* = 35), AD (*n* = 28) and sCJD (*n* = 35) cases (cohort 2). Kruskal-Wallis and Dunn’s post-hoc tests were used for statistical differences estimation. **b** CSF YKL-40 levels in Cognitive Impairment/Dementia (*n* = 26) and sCJD cases (*n* = 14) (cohort 3). Statistical differences were estimated with the Mann–Whitney test.**p < 0.01, ***p < 0.001

**Fig. 9:**
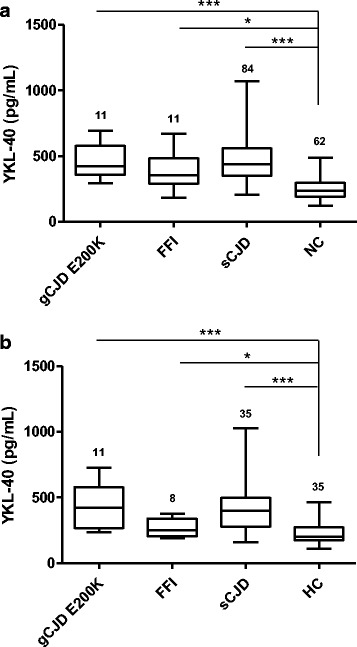
Detection of CSF YKL-40 levels in gCJD E200K and Fatal Familial Insomnia. **a** CSF YKL-40 levels in gCJD E200K, Fatal Familial Insomnia (FFI) and NC (cohort 1). **b** CSF YKL-40 levels in gCJD E200K, Fatal Familial Insomnia (FFI) and HC (cohort 2). Statistical differences were determined with Kruskal-Wallis and Dunn’s post-hoc tests. *p < 0.05, ***p < 0.001

In order to validate the presence of increased YKL-40 levels in sCJD, two independent cohorts were used. The first validation cohort (cohort 2, Additional file 2) included healthy controls (HC), AD and sCJD cases. Compared to HC (226 ± 82 pg/mL), YKL-40 levels were significantly increased in sCJD (411 ± 157 pg/mL, *p* < 0.001) and AD (311 ± 113 pg/mL, *p* < 0.01) (Fig. 8a, cohort 2 Additional file 2). Similar results were obtained for the second validation cohort (cohort 3, Additional file 2), where YKL-40 levels in sCJD were significantly higher (603 ± 198 pg/mL) than those detected in cognitive impairment/dementia cases (358 ± 129 pg/mL, *p* < 0.01) (Fig. 8b, cohort 3 Additional file 2). Finally, we aimed to determine the usefulness of CSF YKL-40 levels in the discrimination of prion disease from genetic etiology. For this purpose, we analysed genetic cases from two cohorts and compared them with their respective controls and sCJD groups. The study included Controls, sCJD, gCJD *PRNP*-E200K and Fatal Familial Insomnia (FFI) (*PRNP*-D178N mutation) cases (cohort 1 and cohort 2, Additional file 2). In both cohorts gCJD cases harbouring the E200K mutation showed similar YKL-40 levels compared to sCJD cases and were statistically different from controls (*p* < 0.001). In contrast, YKL-40 levels in FFI cases were lower than in sCJD cases, but significantly higher compared to control subjects (*p* < 0.05) (Fig. 9a and b and cohort 1 and cohort 2, Additional file 2).

## Discussion

Neuroinflammation in dementia has been identified as a major contributor to disease pathogenesis and as a potentially causative effect of neurodegeneration in chronic profiles [65–67]. However, the precise regional and temporal contribution of glial activation to the pathogenic process is far from understood. Similar to microglial activation, sustained astrocytic activation may lead to a switch from a neuroprotective and neurotrophic to a neurodegenerative phenotype, increasing neuronal vulnerability to chemokines, pro-inflammatory cytokines and reactive oxygen species [68–70]. This is exemplified by proteins such as S100B, which is released from astrocytes with neurotrophic or neurotoxic effects on neurons depending on their concentration and on the astrocytic activation state [71, 72]. In turn, increased expression and secretion of S100B from astrocytes may be reflected in the CSF patterns as reported in traumatic brain injury [73], AD [74, 75] and sCJD [76]. Consequently, astrocytes are turning into a potential target for therapeutic intervention in neurodegenerative diseases associated with an inflammatory component [77], indicating that astrocyte-associated metabolites may be potential biomarkers in biological fluids. Thus, studies on expression patterns and function of secreted, astrocytic-related molecules under pathological or stress conditions are gaining experimental momentum.

The functional role of YKL-40 during neuroinflammation is unclear. However, a neuroprotective effect has been assigned to YKL-40 by an in vivo study, according to which YKL-40 knock-out mice presented more severe neuropathology and more prominent gliosis than their wild-type littermates in a model of controlled cortical impact [78]. These results indicated that YKL-40 modulates the neuroinflammatory response associated with traumatic brain impact (TBI). In agreement with this, YKL-40 knock-out mice present exacerbated experimental autoimmune encephalomyelitis clinical scores accompanied by increased lymphocytic and macrophage infiltrates and gliosis compared to wild-type animals [79], suggesting that YKL-40 is necessary for the proper resolution of inflammation. Importantly, astrocytic YKL-40 expression is under the control of microglial stimuli since YKL-40 transcription, accompanied by morphological changes and increased migratory capacity characteristic of reactive gliosis, is induced by the macrophage-released pro-inflammatory mediators Interleukin-1β (IL-1β) and tumor necrosis factor-α [22]. Interestingly, both cytokines are highly expressed in the microglia of TBI, sCJD, AD and related mouse models [40, 41, 80], suggesting a cross-talk between microglial and astrocytic activation in the regulation of YKL-40-mediated functions in chronic inflammatory profiles associated with neurodegeneration. These data are supported by the finding that YKL-40 expression correlates with the expression of IL-1β and Interleukin-6 and that both cytokines are able to synergistically up-regulate YKL-40 expression in primary astrocytes in vitro in a STAT3 dependent-manner [81], which in turn, has been seen to be activated in the FC of sCJD brains [40]. In this regard, it has been recently shown that a subtype of reactive astrocytes present in neurodegenerative diseases is induced by secreted cytokines from activated microglia [82], reinforcing the suggested role of astrocyte-microglia cross-talk during neurodegeneration [83, 84].

Aiming at a better characterization of YKL-40’s role in neurodegenerative disorders, we studied its mRNA and protein levels and its expression in different brain regions in several neurodegenerative conditions. Astrocytic YKL-40 overexpression was prominent in both sCJD and AD, in agreement with the well-known chronic inflammatory profile of both diseases [38]. Most of the YKL-40+ astrocytes were stained with GFAP antibodies, both in sCJD and AD. However, a small population of astrocytes with single labelling for one of the two markers was also detected, in agreement with the positive but moderate correlation between GFAP and YKL-40 expression levels detected in sCJD.

Further, the absence of YKL-40 overexpression in DLB was in line with the corresponding low inflammatory profile in DLB [42, 85]. Disease-specific differences were not restricted to expression levels, but also included regional-dependent regulation. Indeed, we demonstrated that YKL-40 overexpression in the cerebellum is restricted to sCJD and absent in AD.

Data obtained from prion models suggest a role of YKL-40 at pre-clinical and early stages of the disease. In RML infected mice, early prion pathology including microglia activation, astrogliosis and PrPSc deposition could be first detected in the thalamus, whereas hippocampus and cortex still remain unchanged at the microscopic level. At clinical disease stages, hippocampus and cerebellum show widespread signs of prion pathology. This is in line with our detection of YKL-40 immunoreactivity in RML infected mice, which was abundant in the thalamus only at preclinical time points, but was more widespread, including hippocampus, at clinical prion disease stages. This is supported by the elevated levels of YKL-40 in cortical region of the sCJD model at pre-clinical disease stage.

Incorporation of new CSF biomarkers in clinical practice not only improves current diagnostic approaches across the disease continuum, but also provides new tools for prognosis and evaluation of therapeutic efficacy once disease-modifying treatments become available [86–88]. Therefore, peripheral biomarkers mirroring specific disease hallmarks such as neuronal/synaptic damage, neuroinflammation and the presence of misfolded proteins will become useful to evaluate therapeutic approaches. In this regard, we demonstrated that CSF YKL-40 patterns resemble expression levels in brain tissue in a disease-specific manner. Additionally, we detected elevated YKL-40 in the brain of early AD (Braak stages I-III). Although data regarding elevated CSF YKL-40 in early stages of AD are contradictory [24, 25, 89, 90], our results support the presence of increased astrocytic YKL-40 expression, together with a role of astrocytosis, in early AD pathogenesis [91]. Similarly, we detected increased YKL-40 in the brain of an AD mouse model and in experimental models of prion diseases at pre-clinical stages, supporting the presence of astrocytic alterations before clinical onset and the usefulness of YKL-40 quantification in the detection of early pathological changes associated with neuroinflammation. Interestingly increase in YKL-40 at pre-clinical stages (120 dpi) of the sCJD mice model occurs when PrPres, but not synaptic damage, is already detectable [92] [Llorens et al. 2017, submitted], supporting the hypothesis that neuroinflammation may precede neuronal dysfunction.

Additionally, it has recently been shown that in MCI and pre-AD stages, APOE ε4 carriers display higher CSF levels of YKL-40 but not of a marker of microglial activation, sTREM2 (soluble triggering receptor expressed on myeloid cells 2), supporting a role of APOE ε4 in the regulation of astroglial response in AD pathology [93]. However, CSF from pre-sCJD stages is not available to rule out a potential role of CSF YKL-40 as pre-clinical marker of prion diseases. In this regard, use of in vivo prion models not only validates regional alterations observed in human tissue, but also supports utilization of humanized sCJD mouse models in the understanding of human pathogenesis at pre-clinical disease stages, since scrapie infected animals present strain-specific regional alterations that differ from those detected in human and mouse sCJD samples.

An interesting finding of our study is the detection of increased CSF YKL-40 levels in FFI, supporting the idea that astrogliosis is a common hallmark in FFI brain tissue even in regions with no major pathological involvement [94, 95]. In contrast, CSF surrogate markers of neuronal damage are less sensitive in detecting FFI cases [44, 96] as neuronal damage is restricted to thalamic nuclei. Elevated YKL-40 levels in gCJD-E200K patients were in line with the similar diagnostic parameters between sCJD and gCJD-E200K for other prion biomarkers [44, 96], due to the common clinico-pathological spectrum of gCJD-E200K and sCJD cases [97, 98]. Despite high CSF YKL-40 levels were found in sCJD and AD, increased YKL-40 levels have also been detected in other neurological and psychiatric conditions such as brain concussions [99], acute ischemic stroke [100], suicidal ideation [101] and bipolar disorder [102], underlining that astrocytic activation is not restricted to neurodegenerative conditions and that neuroinflammation may play a more prominent and common role in the etiology of neurological diseases.

Conflicting data have been reported on the correlation of YKL-40 with markers of neurodegeneration [23, 25, 31, 32, 89] as well as with cognitive decline [28, 31, 36, 89]. The distinct correlations between different surrogate disease markers and cognitive decline suggest that dementia hallmarks may reflect different aspects of neurodegeneration processes which, although potentially associated, may provide independent information about the pathological state of the brain tissue. In this regard, it has been proven that, in early AD, CSF YKL-40 is associated with a cerebral structural signature distinct from that related to p-tau neurodegeneration [28], even when a positive correlation between the levels of the two biomarkers in the CSF is reported [23–25, 34].

For sCJD, the positive correlation between CSF tau and YKL-40 suggests an association between axonal damage and neuroinflammation. Indeed, it is well known that inflammatory patterns in sCJD correlate with the severity of the neuropathological lesions [103]. In contrast, no correlation was detected between age and disease duration, most likely due to the very short disease duration of sCJD cases (mean disease duration = 7.0 ± 5.9 months).

## Conclusions

Here we report, for the first time, that higher YKL-40 levels are detectable in the brain and CSF in prion diseases. Further we show that CSF YKL-40 presents a good diagnostic accuracy in the discrimination of sCJD from NC. While YKL-40 is also elevated in AD, it failed discriminating AD, VaD and DLB/PDD cases (AUC < 0.8). In this regard, caution should be taken when interpreting elevated CSF YKL-40 levels in the diagnostic context of neurodegenerative dementias. However, since CSF YKL-40 resembles astrocytic activation in brain tissue we speculate that YKL-40 quantification may be useful for the assessment of neuroinflammatory profiles during therapeutic intervention.

## Additional files

Additional file 1: Summary of human brain cases and regions analyzed with qPCR, western blot and immunohistochemical analysis. Number of cases, age (mean ± SD) and gender (number of females and males) are indicated. (TIFF 93 kb)

Additional file 2: Summary of CSF samples from the three independent cohorts used in this study. Sample group, number of cases, age (mean ± SD), tau levels (pg/mL), Aβ42 levels (pg/mL), 14–3-3 positive cases and YKL-40 levels (pg/mL) are indicated. (TIFF 147 kb)

Additional file 3: YKL-40 mRNA expression in sCJD and AD using GUSB as housekeeping gene. Validation of alterations in YKL-40 mRNA in sCJD (A) and AD (B) cases using GUSB as qPCR housekeeping gene. (TIFF 59 kb)

Additional file 4: YKL-40-positive astrocytes associated to PrP amyloid plaques. Immunofluorescence images obtained from double-labeling staining with GFAP (A) and YKL-40 (B) (green) and PrP (red) antibodies in the cortex of sCJD. Individual channels as well as merge images are shown for two different cortical areas. (TIFF 768 kb)

Additional file 5: Quantification of YKL-40 and GFAP overlap in sCJD and AD cases. Immunofluorescence images obtained from double-labelling staining with GFAP (red) and YKL-40 (green) antibodies in the hippocampus of sCJD (*n* = 3) (A) and AD V (n = 3) (C) cases. Individual channels as well as merge images are shown for two different cortical areas (one cortical image per CJD or AD patient) of two different patients. Quantifications of GFAP+/YKL-40+ (YKL-40-GFAP), GFAP+/YKL-40- (GFAP) and GFAP−/YKL-40+ (YKL-40) astrocytes are shown. Statistical significance differences were detected between YKL-40-GFAP and YKL-40, GFAP groups; ###*p* < 0.001 and ***p < 0.001, respectively. (TIFF 5257 kb)

Additional file 6: YKL-40 expression in AD. (A) YKL-40 expression in astrocytes in the dentate gyrus of AD. (B) Immunohistochemical analysis of YKL-40+ astrocytes surrounding β-amyloid plaques in AD cases. (TIFF 989 kb)

Additional file 7: YKL-40 positive astrocytes associated to β-amyloid plaques. Confocal Z-stack images obtained from double-labelling staining with YKL-40 (green) and β-amyloid (red) antibodies in the hippocampus of AD tissue. Z-stacks from representative senile β-amyloid plaque (A) and diffuse amyloid plaque (B) is shown. DRAQ5 staining is shown in blue. Individual channels as well as merge images are shown. Distance between-Z Stacks sections = 0.5 μm. (ZIP 2287 kb)

Additional file 8: YKL-40 expression in 5xFAD mice. (A) Western blot analysis of YKL-40 and GFAP in the cortex of 3-month-old control and 5xFAD animals. Four animals per group were analyzed. GAPDH was used for normalization. Numbers represent the summary of densitometric analysis. Unpaired t-test was used for statistical differences estimation. (B) Immunofluorescence analysis of YKL-40 (green) in the cerebellar cortex and hippocampus of 10-month-old 5xFAD mice. Nuclei were stained with DAPI (blue). Three animals per group and three sections per animal were analyzed. (TIFF 235 kb)
