# Supplementary figures and images for: YKL-40 in the brain and cerebrospinal fluid of neurodegenerative dementias
Source: Mol Neurodegener. 2017 Nov 10;12:83. doi: 10.1186/s13024-017-0226-4 (PMC5681777; doi:10.1186/s13024-017-0226-4)

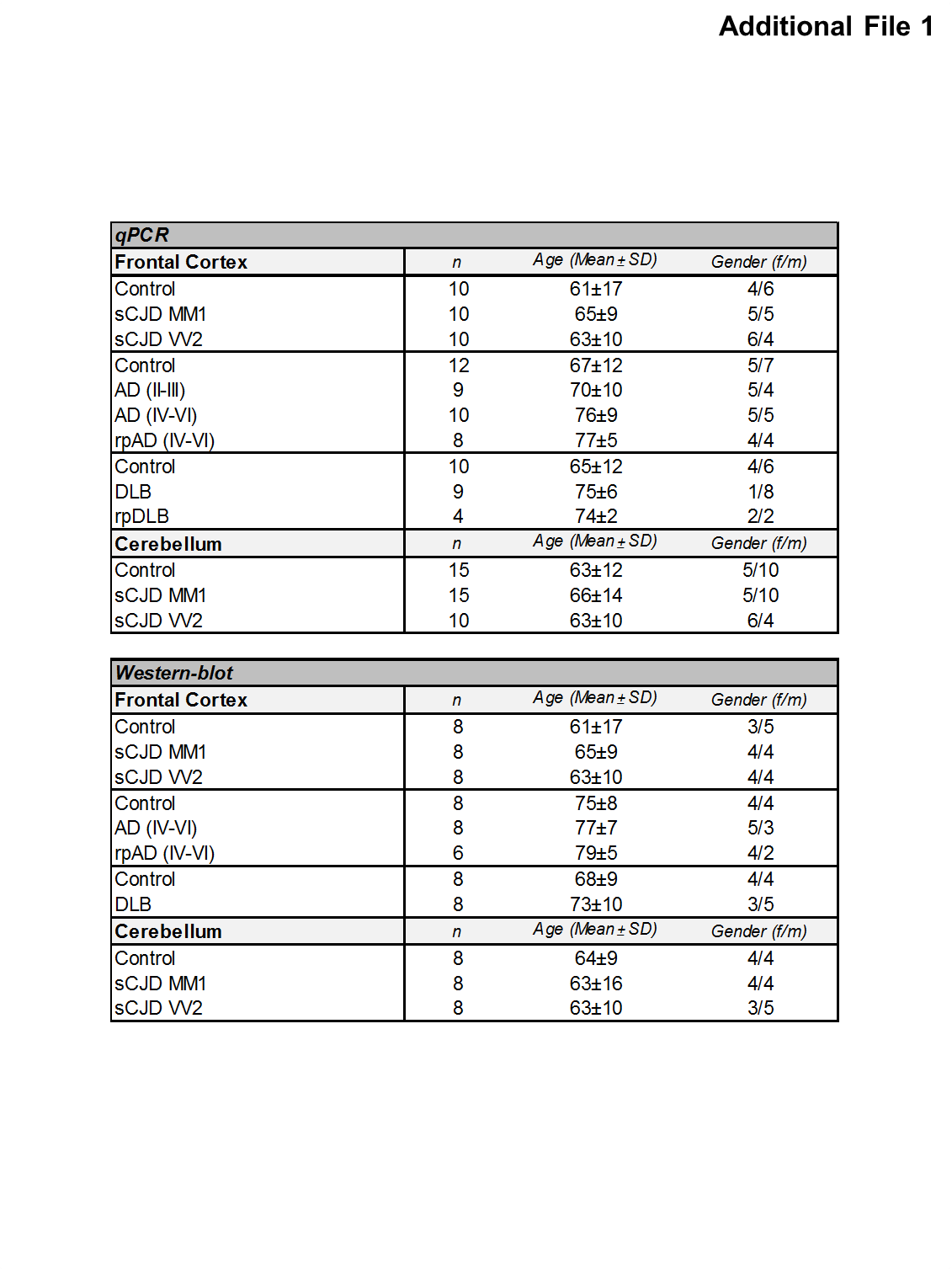

Supplement: Supplementary file 1 — Summary of human brain cases and regions analyzed with qPCR, western blot and immunohistochemical analysis. Number of cases, age (mean ± SD) and gender (number of females and males) are indicated. (TIFF 93 kb) [file 13024_2017_226_MOESM1_ESM.tif]

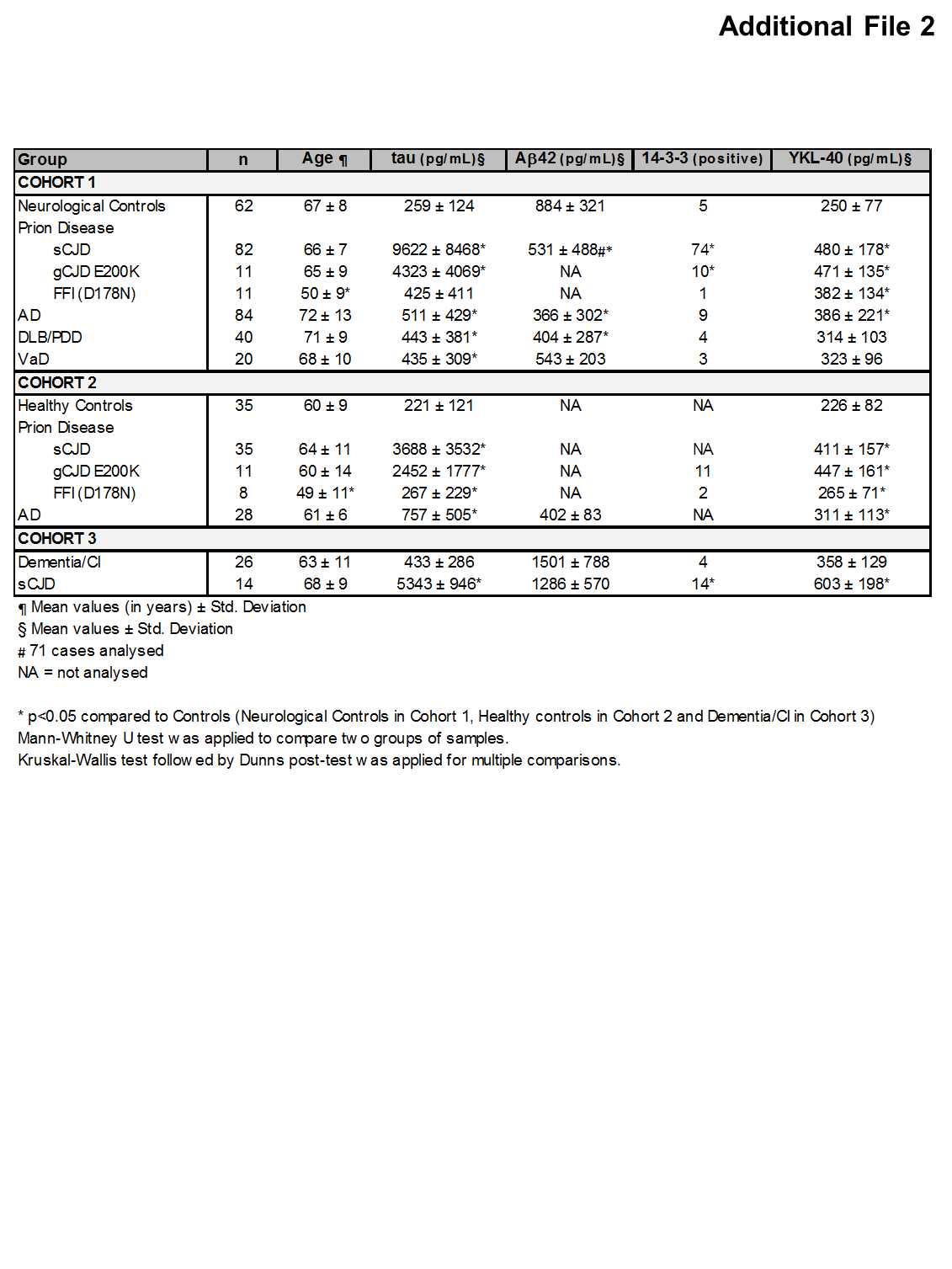

Supplement: Supplementary file 2 — Summary of CSF samples from the three independent cohorts used in this study. Sample group, number of cases, age (mean ± SD), tau levels (pg/mL), Aβ42 levels (pg/mL), 14–3-3 positive cases and YKL-40 levels (pg/mL) are indicated. (TIFF 147 kb) [file 13024_2017_226_MOESM2_ESM.tif]

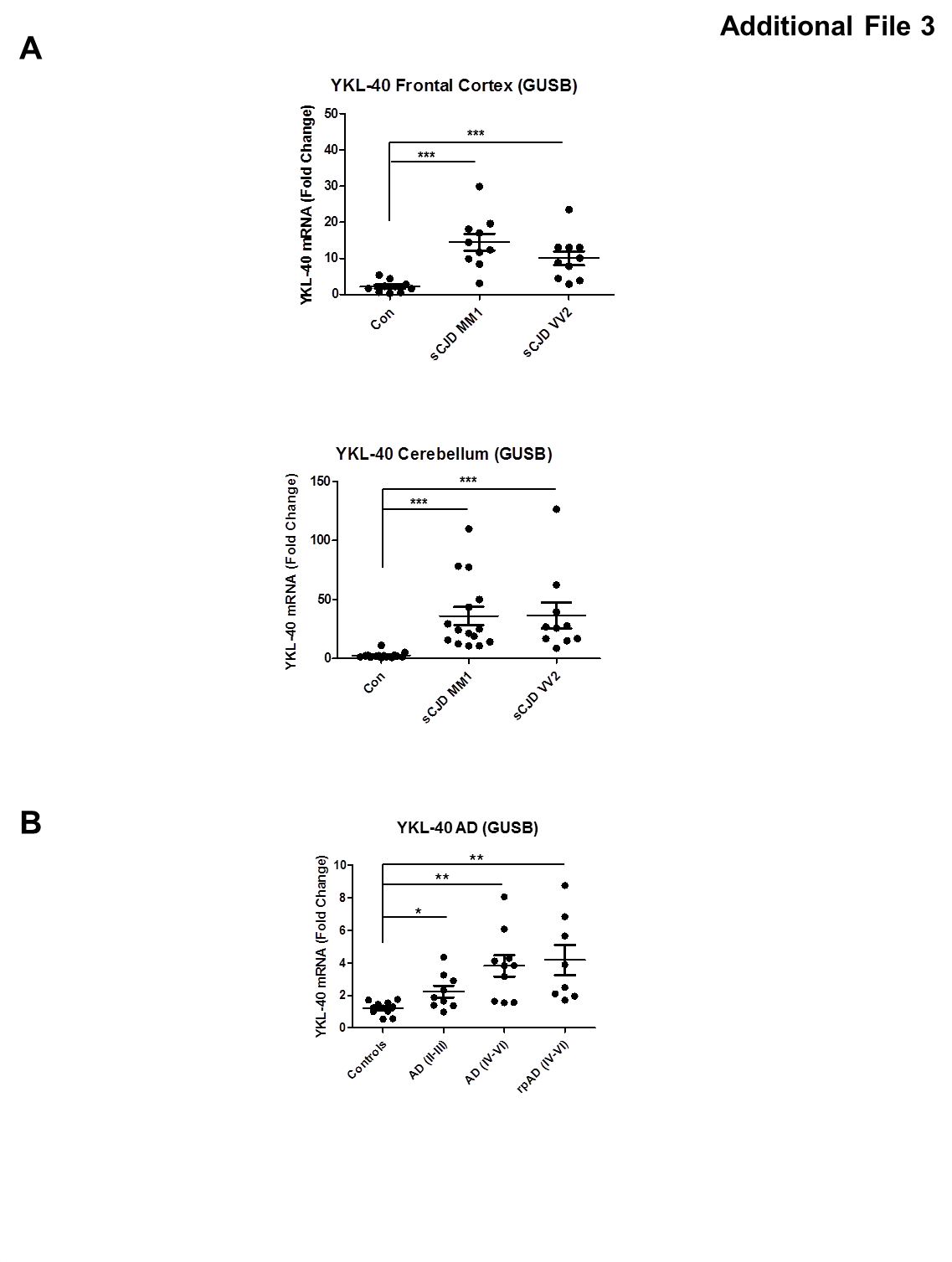

Supplement: Supplementary file 3 — YKL-40 mRNA expression in sCJD and AD using GUSB as housekeeping gene. Validation of alterations in YKL-40 mRNA in sCJD (A) and AD (B) cases using GUSB as qPCR housekeeping gene. (TIFF 59 kb) [file 13024_2017_226_MOESM3_ESM.tif]

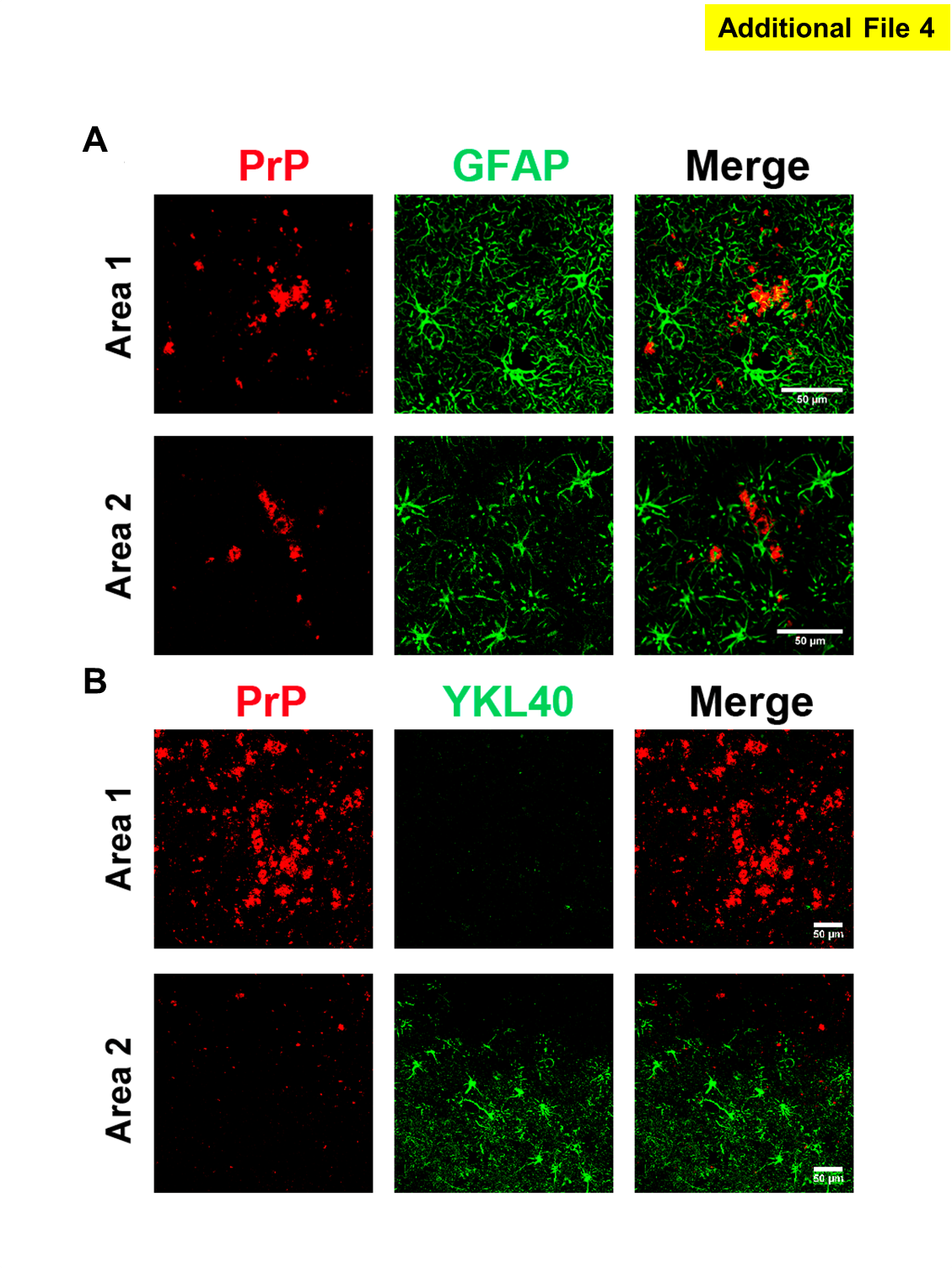

Supplement: Supplementary file 4 — YKL-40-positive astrocytes associated to PrP amyloid plaques. Immunofluorescence images obtained from double-labeling staining with GFAP (A) and YKL-40 (B) (green) and PrP (red) antibodies in the cortex of sCJD. Individual channels as well as merge images are shown for two different cortical areas. (TIFF 768 kb) [file 13024_2017_226_MOESM4_ESM.tif]

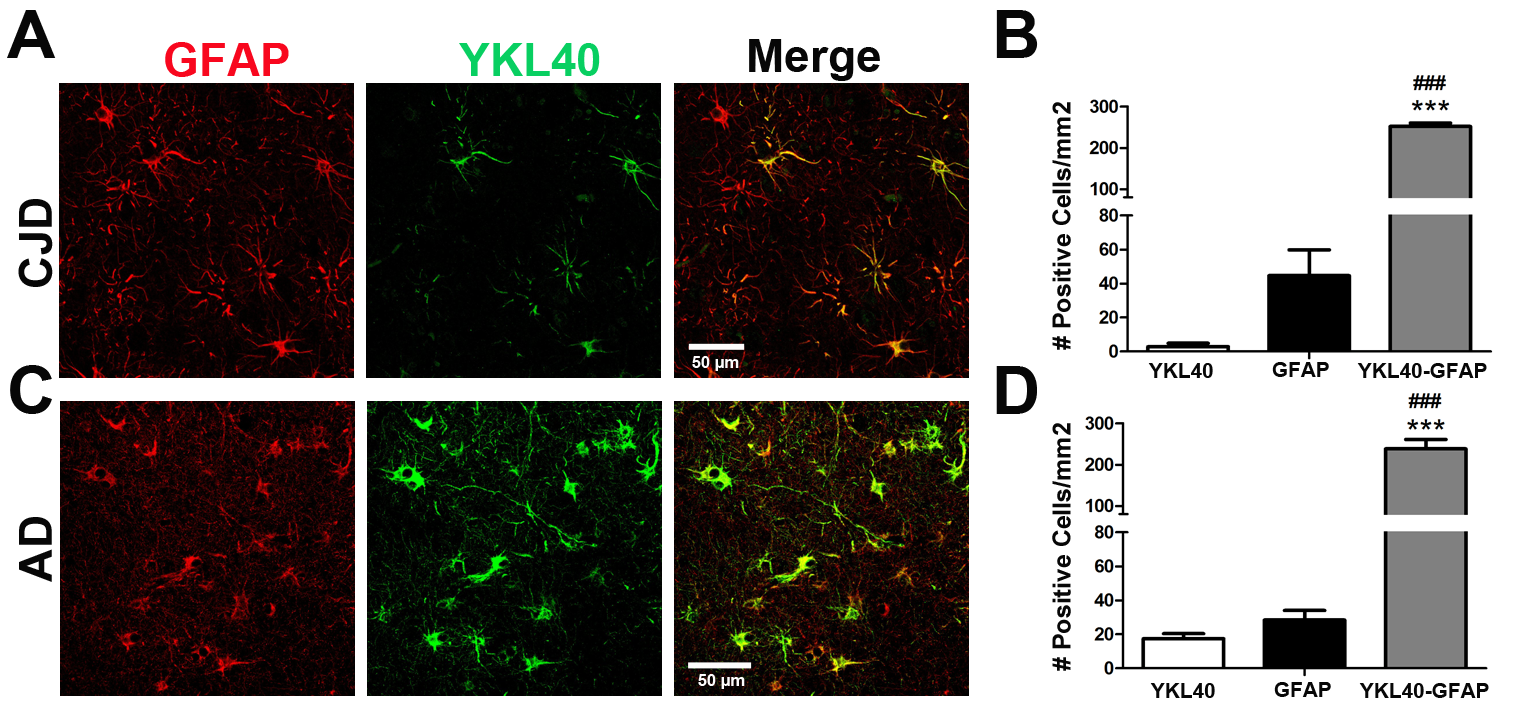

Supplement: Supplementary file 5 — Quantification of YKL-40 and GFAP overlap in sCJD and AD cases. Immunofluorescence images obtained from double-labelling staining with GFAP (red) and YKL-40 (green) antibodies in the hippocampus of sCJD (n = 3) (A) and AD V (n = 3) (C) cases. Individual channels as well as merge images are shown for two different cortical areas (one cortical image per CJD or AD patient) of two different patients. Quantifications of GFAP+/YKL-40+ (YKL-40-GFAP), GFAP+/YKL-40- (GFAP) and GFAP−/YKL-40+ (YKL-40) astrocytes are shown. Statistical significance differences were detected between YKL-40-GFAP and YKL-40, GFAP groups; ###p < 0.001 and ***p < 0.001, respectively. (TIFF 5257 kb) [file 13024_2017_226_MOESM5_ESM.tif]

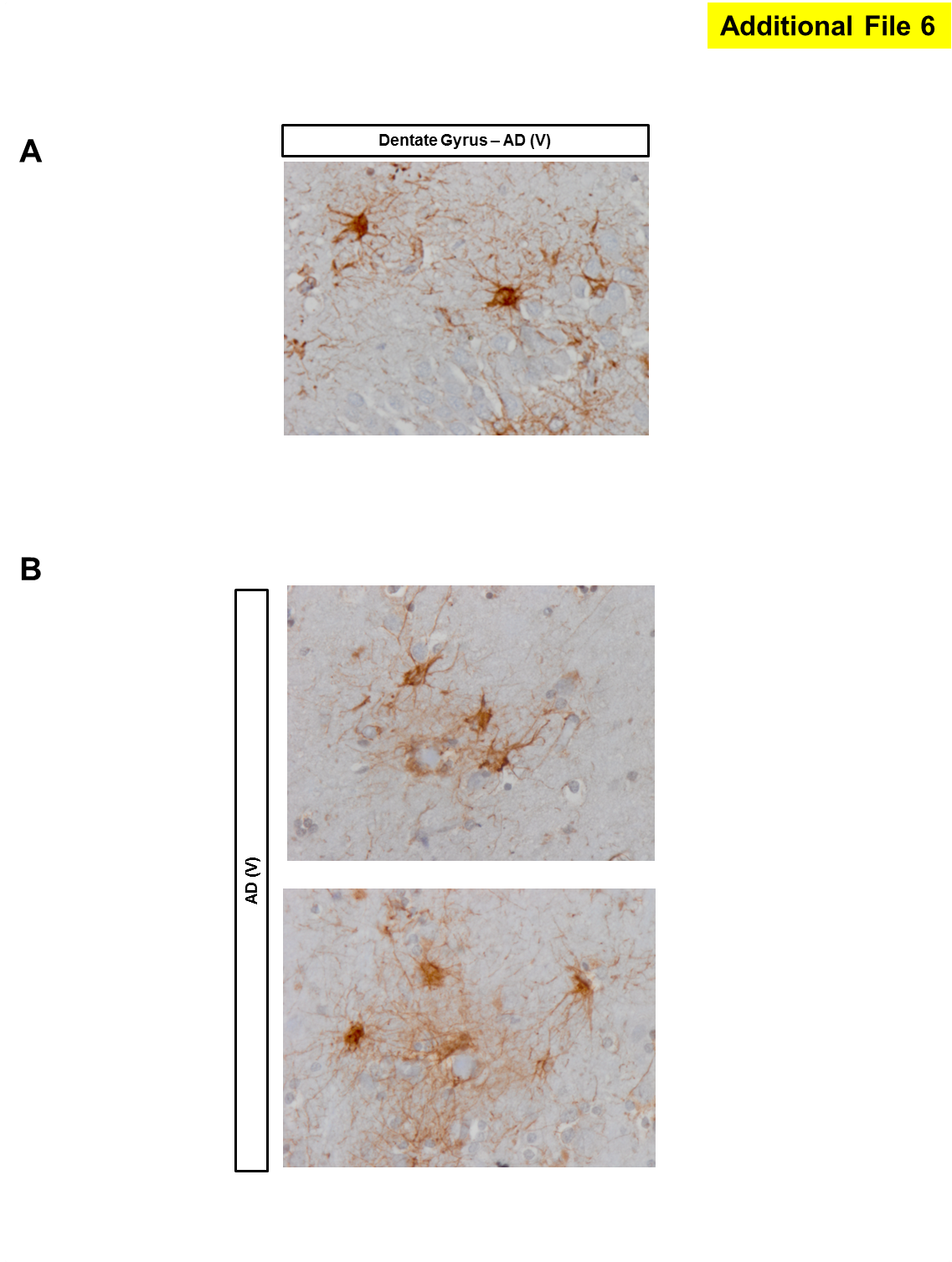

Supplement: Supplementary file 6 — YKL-40 expression in AD. (A) YKL-40 expression in astrocytes in the dentate gyrus of AD. (B) Immunohistochemical analysis of YKL-40+ astrocytes surrounding β-amyloid plaques in AD cases. (TIFF 989 kb) [file 13024_2017_226_MOESM6_ESM.tif]

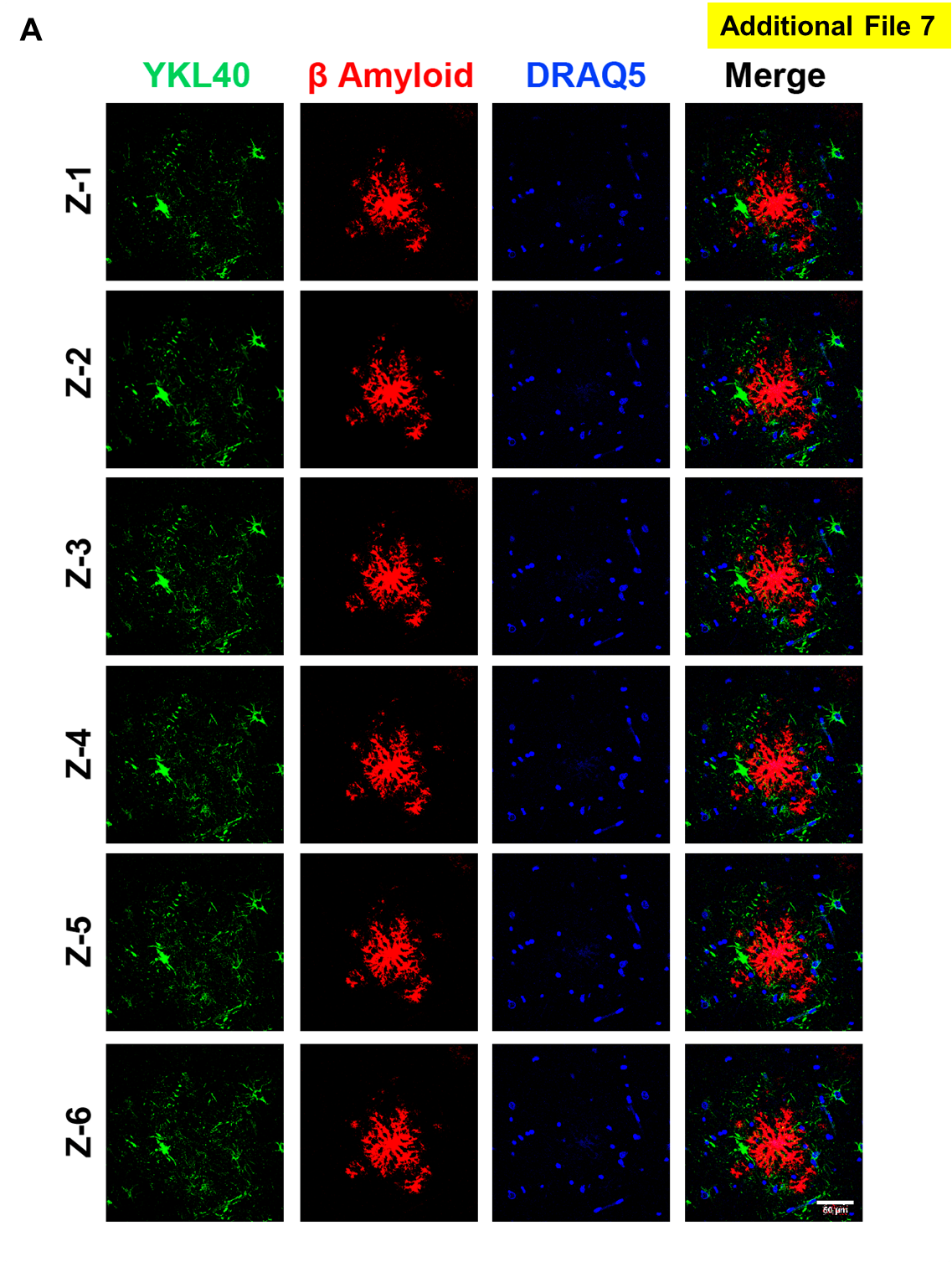

Supplement: Supplementary file 7 — YKL-40 positive astrocytes associated to β-amyloid plaques. Confocal Z-stack images obtained from double-labelling staining with YKL-40 (green) and β-amyloid (red) antibodies in the hippocampus of AD tissue. Z-stacks from representative senile β-amyloid plaque (A) and diffuse amyloid plaque (B) is shown. DRAQ5 staining is shown in blue. Individual channels as well as merge images are shown. Distance between-Z Stacks sections = 0.5 μm. (ZIP 2287 kb) [file 13024_2017_226_MOESM7_ESM.zip › Additional File 7A.tif]

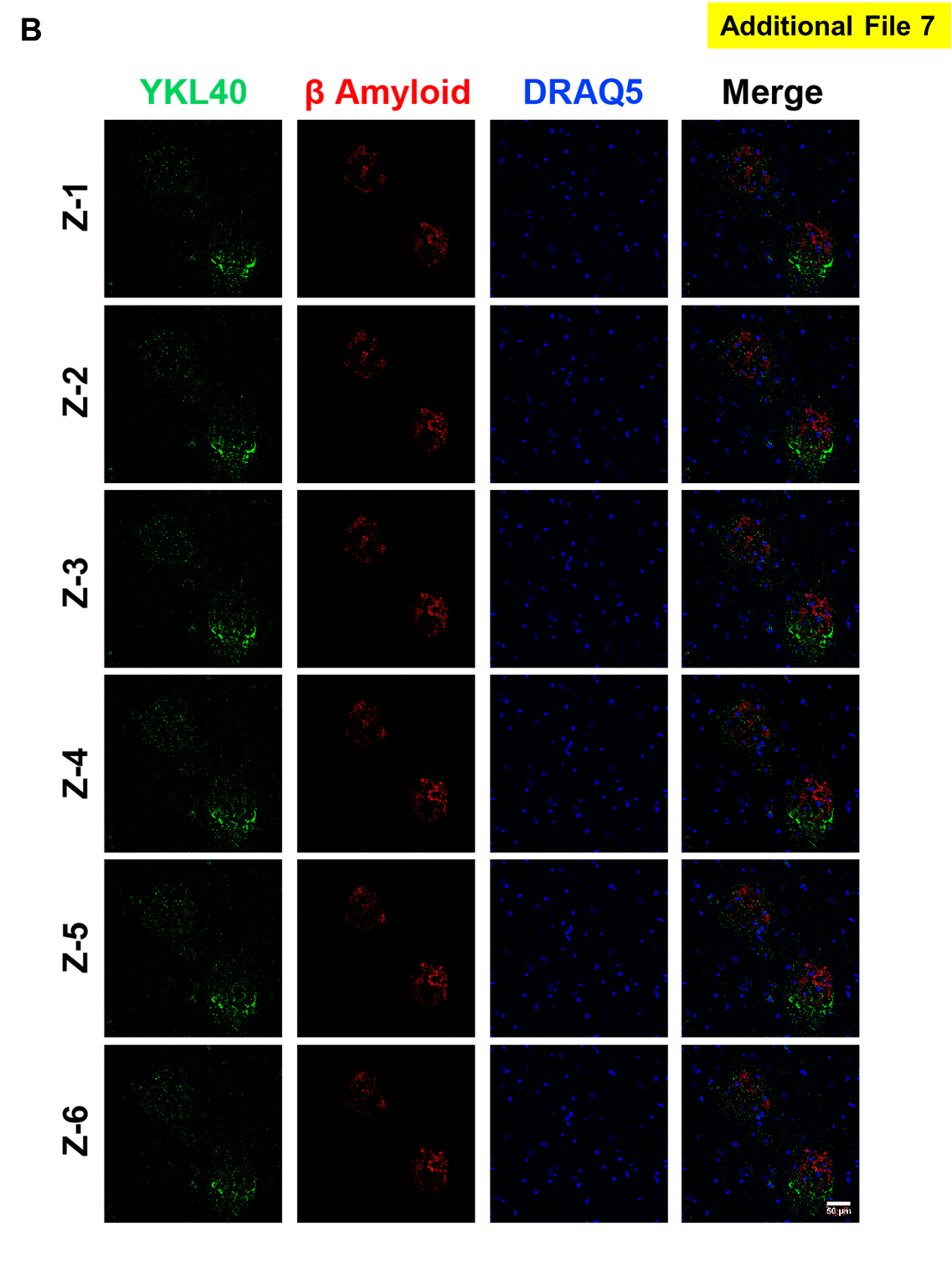

Supplement: Supplementary file 7 — YKL-40 positive astrocytes associated to β-amyloid plaques. Confocal Z-stack images obtained from double-labelling staining with YKL-40 (green) and β-amyloid (red) antibodies in the hippocampus of AD tissue. Z-stacks from representative senile β-amyloid plaque (A) and diffuse amyloid plaque (B) is shown. DRAQ5 staining is shown in blue. Individual channels as well as merge images are shown. Distance between-Z Stacks sections = 0.5 μm. (ZIP 2287 kb) [file 13024_2017_226_MOESM7_ESM.zip › Additional File 7B.tif]

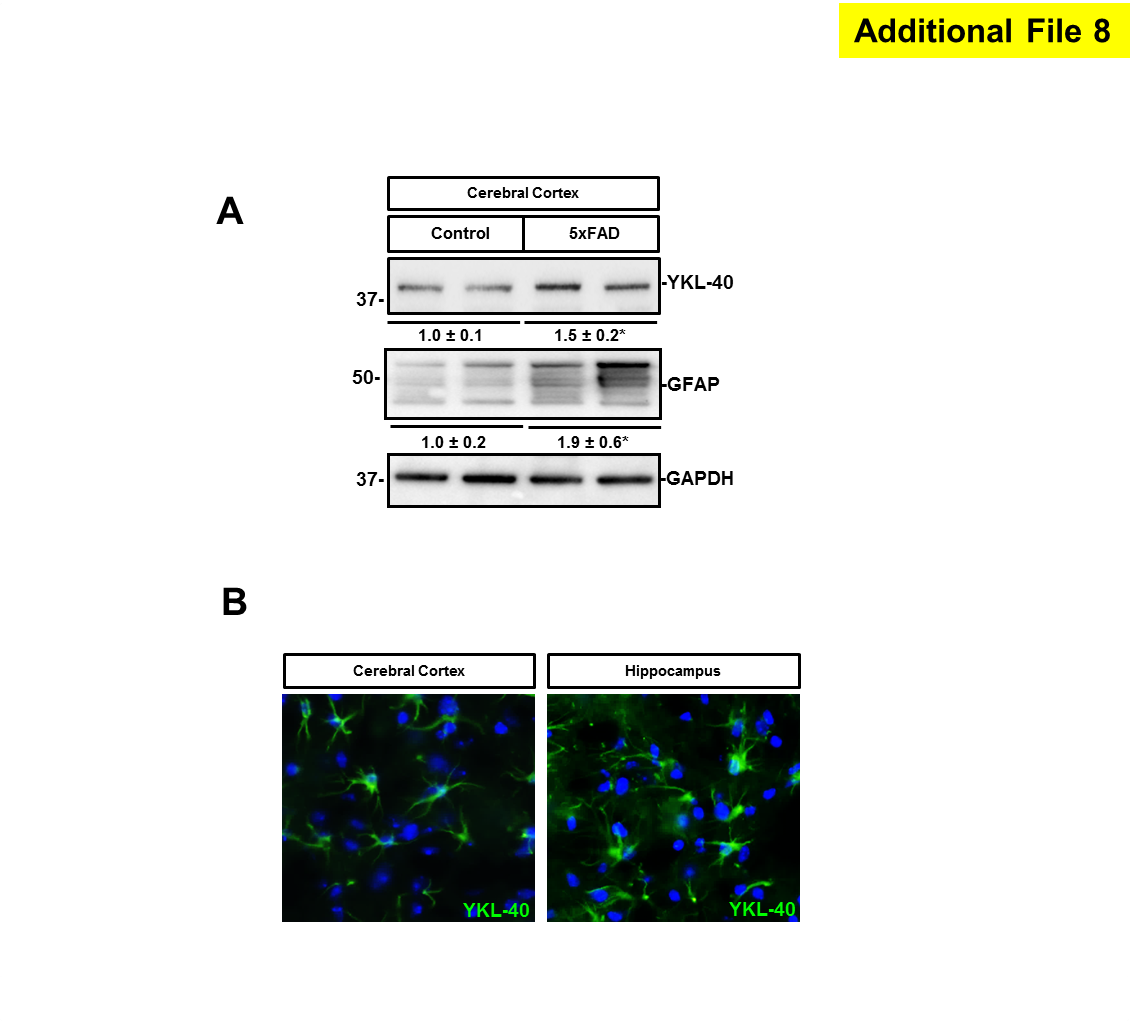

Supplement: Supplementary file 8 — YKL-40 expression in 5xFAD mice. (A) Western blot analysis of YKL-40 and GFAP in the cortex of 3-month-old control and 5xFAD animals. Four animals per group were analyzed. GAPDH was used for normalization. Numbers represent the summary of densitometric analysis. Unpaired t-test was used for statistical differences estimation. (B) Immunofluorescence analysis of YKL-40 (green) in the cerebellar cortex and hippocampus of 10-month-old 5xFAD mice. Nuclei were stained with DAPI (blue). Three animals per group and three sections per animal were analyzed. (TIFF 235 kb) [file 13024_2017_226_MOESM8_ESM.tif]
